# A Promising New Model: Establishment of Patient‐Derived Organoid Models Covering HPV‐Related Cervical Pre‐Cancerous Lesions and Their Cancers

**DOI:** 10.1002/advs.202302340

**Published:** 2024-01-16

**Authors:** Bai Hu, Renjie Wang, Di Wu, Rui Long, Junpeng Fan, Zhe Hu, Xingyuan Hu, Ding Ma, Fang Li, Chaoyang Sun, Shujie Liao

**Affiliations:** ^1^ Department of Gynecology and Obstetrics Tongji Hospital Tongji Medical College Huazhong University of Science and Technology Wuhan Hubei 430030 China; ^2^ National Clinical Research Center for Obstetrics and Gynecology Cancer Biology Research Center (Key Laboratory of the Ministry of Education) Tongji Hospital Tongji Medical College Huazhong University of Science and Technology Wuhan Hubei 430030 China; ^3^ Department of Obstetrics and Gynecology Shanghai East Hospital School of Medicine Tongji University Shanghai 200120 China

**Keywords:** cervical cancer, coculture, human papillomavirus, organoids, squamous intraepithelial lesion

## Abstract

The lack of human‐derived in vitro models that recapitulate cervical pre‐cancerous lesions has been the bottleneck in researching human papillomavirus (HPV) infection‐associated pre‐cancerous lesions and cancers for a long time. Here, a long‐term 3D organoid culture protocol for high‐grade squamous intraepithelial lesions and cervical squamous cell carcinoma that stably recapitulates the two tissues of origin is described. Originating from human‐derived samples, a small biobank of cervical pre‐tumoroids and tumoroids that faithfully retains genomic and transcriptomic characteristics as well as the causative HPV genome is established. Cervical pre‐tumoroids and tumoroids show differential responses to common chemotherapeutic agents and grow differently as xenografts in mice. By coculture organoid models with peripheral blood immune cells (PBMCs) stimulated by HPV antigenic peptides, it is illustrated that both organoid models respond differently to immunized PBMCs, supporting organoids as reliable and powerful tools for studying virus‐specific T‐cell responses and screening therapeutic HPV vaccines. In this study, a model of cervical pre‐cancerous lesions containing HPV is established for the first time, overcoming the bottleneck of the current model of human cervical pre‐cancerous lesions. This study establishes an experimental platform and biobanks for in vitro mechanistic research, therapeutic vaccine screening, and personalized treatment for HPV‐related cervical diseases.

## Graphical abstract

Here, a long‐term 3D organoid culture protocol for high‐grade squamous intraepithelial lesions and cervical squamous cell carcinoma that stably recapitulates the two tissues of origin is described. This study establishes an experimental platform and biobanks for in vitro mechanistic research, vaccine development, targeted therapy research, and personalized treatment of human papillomavirus‐related cervical diseases.

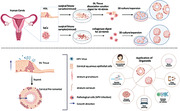

## 1 Introduction

Cervical cancer is one of the most common gynecological tumors.^[^ ^1^ ^]^ More than 604 000 women are diagnosed with cervical cancer annually worldwide, resulting in over 342 000 deaths.^[^ ^2^ ^]^ Although the incidence of cervical cancer has greatly reduced since the widespread use of human papillomavirus (HPV) vaccines and cervical cancer screening, cervical cancer remains the second most common gynecological tumor in developing countries.^[^ ^3^ ^]^ The most prevalent subtype of cervical cancer is squamous cell carcinoma (SqCa), which accounts for 70% of all cases.^[^ ^4^ ^]^ Before the late‐stage presentation of cervical SqCa, the disease malignant transformation progression from HPV infection—low‐grade squamous intraepithelial lesion (LSIL)—high‐grade squamous intraepithelial lesion (HSIL) to cervical SqCa lasts for 10 years or more.^[^ ^5^ ^]^ Therefore, a better understanding of the premalignant state preceding neoplasia is required. The development of accurate premalignancy models can address this unmet need by providing prevention and early intervention strategies. Furthermore, these models can help define the key elements of gene interactions that govern the premalignancy‐to‐cancer transition.

Currently, research on cervical premalignancy and cervical SqCa depends on a finite number of models, such as cell lines, K14‐HPV16 transgenic mice, and patient‐derived tumor xenotransplantation (PDX). Cell lines such as Ect1/E6E7, HeLa, and Siha have been established for a long time, and their phenotypes and genetic information differ significantly from those of their parents after multiple passages, and only contain a small portion of tumor genetic information. In particular, the cervical pre‐cancerous lesion cell line Ect1/E6E7, which is widely used as a model for HSIL disease, was immortalized by the introduction of HPV‐derived oncogenes E6 or E7,^[^ ^6^ ^]^ lacking a complete HPV genome and could not reflect the true situation of HPV‐related disease. The corresponding animal models of HPV‐related cervical diseases include K14‐HPV16 transgenic mice and PDX. K14‐HPV16 transgenic mice, the only animal model of HSIL at present, mimic the pathological process of HSIL by transferring the HPV16 gene into mice; however, its non‐HPV‐infected state and non‐human cellular background make it differ greatly from the real HSIL situation. Another well‐known model is the animal PDX, which is limited because of large species differences, long culture periods, high costs, complex operations, and uncontrollable factors.^[^ ^7^ ^]^ Therefore, there is an urgent need to establish a humanized high‐fidelity squamous intraepithelial lesion model.

Organoids are an emerging technology that culture stem cells in vitro in a 3D environment, recapitulating the structures and molecular features of the tissue of origin.^[^ ^8^ ^]^ In addition to 2D cell culture and animal models, organoids have the advantages of short modeling times, high modeling success rates, and good fidelity. Patient‐derived organoids (PDO) maintain the genetic and morphological heterogeneity of tumors and have outstanding value in studying tumorigenesis, progression,^[^ ^9^ ^]^ drug resistance,^[^ ^10^ ^]^ new drug screening,^[^ ^11^ ^]^ personalized therapy,^[^ ^12^ ^]^ and other aspects.^[^ ^7^, ^10^ ^]^ Lohmussaar et al. established long‐term human organoid cultures from healthy ectocervical tissues and SqCa.^[^ ^13^ ^]^

However, an organoid model of HSIL has not yet been developed. In this study, we established innovative PDO models covering the occurrence and development of SqCa in the context of HPV, that is, organoid models simulating two disease states: pre‐cervical and cervical cancer. These organoids may contribute to the understanding of the mechanism of HPV and host interactions, the transition from HSIL to SqCa, and research on new treatments for cervical pre‐cancer/cancer.

## 2 Results

### 2.1 Establishment of Long‐Term Expandable Organoids from HSIL and Cervical SqCa Lesions

**Figure 1:**
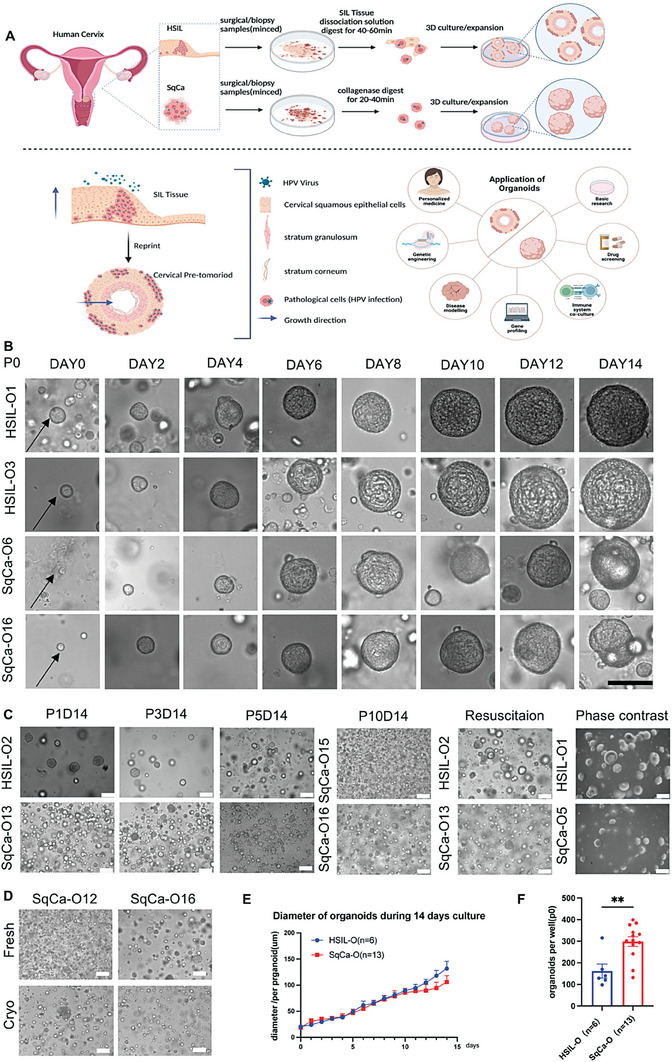
Expandable organoids established from HSIL and SqCa tissues. A) Diagram of organoid culture process. High‐grade squamous intraepithelial lesion (HSIL) and squamous carcinoma of the cervix (SqCa) samples were obtained from patients undergoing surgery or biopsy (patients’ information detailed in Table S1, Supporting Information) and were processed as described in the Experimental Section. HSIL and SqCa samples were treated with SIL Tissue dissociation solution (composed of collagenase I, Dispase II, and DNase I) or collagenase I, respectively. The cells or groups of cells were seeded into basement membrane extract (BME) and cultured in the appropriate medium. Following this protocol, organoids could be derived with 84.6 and 83.3% success rates in the HSIL‐organoid and SqCa‐organoid lines, respectively. After establishing the SIL tissue as the organoid, we confirmed that the SIL organoid was a copy of the tissue, with a stereo‐spherical form of multilayered squamous epithelium growing from the outside to the inside, while retaining HPV‐infected pathological cervical cells, which were named cervical pre‐tumoroids. Organoids have various applications such as basic research, drug screening, immune system coculture, new antigen screening, gene profiling, disease modeling, genetic engineering, and personalized medicine. B) Brightfield images of HSIL‐O and SqCa‐O in their respective medium over a 2‐week course after seeding (P0). Representative images from three independent experiments are shown. Scale bar, 100 µm. C) Brightfield images of different passages demonstrating the long‐term expansion, growth vigor after frozen storage and recovery, and morphology under the contrast microscope of HSIL‐O and SqCa‐O. Representative images of three independent experiments are shown. Scale bars, 100 µm in the overview. D) Organoids established by cells or groups of cells in fresh and cryo states. Scale bars, 100 µm in the overview. E) During the culture process, we monitored the diameter of a single organoid of HSIL‐O (*n* = 6) and SqCa‐O (*n* = 13) for 14 days (P0). Error bars represent the SEM. F) The formation efficiency of HSIL‐O and SqCa‐O (horizontal lines and error bars show the mean number of organoids formed ± SEM. of *n* = 6 (HSIL‐O) and *n* = 13 (SqCa‐O) biologically independent experiments. **P < 0.01; unpaired *t*‐test).

**Figure 2:**
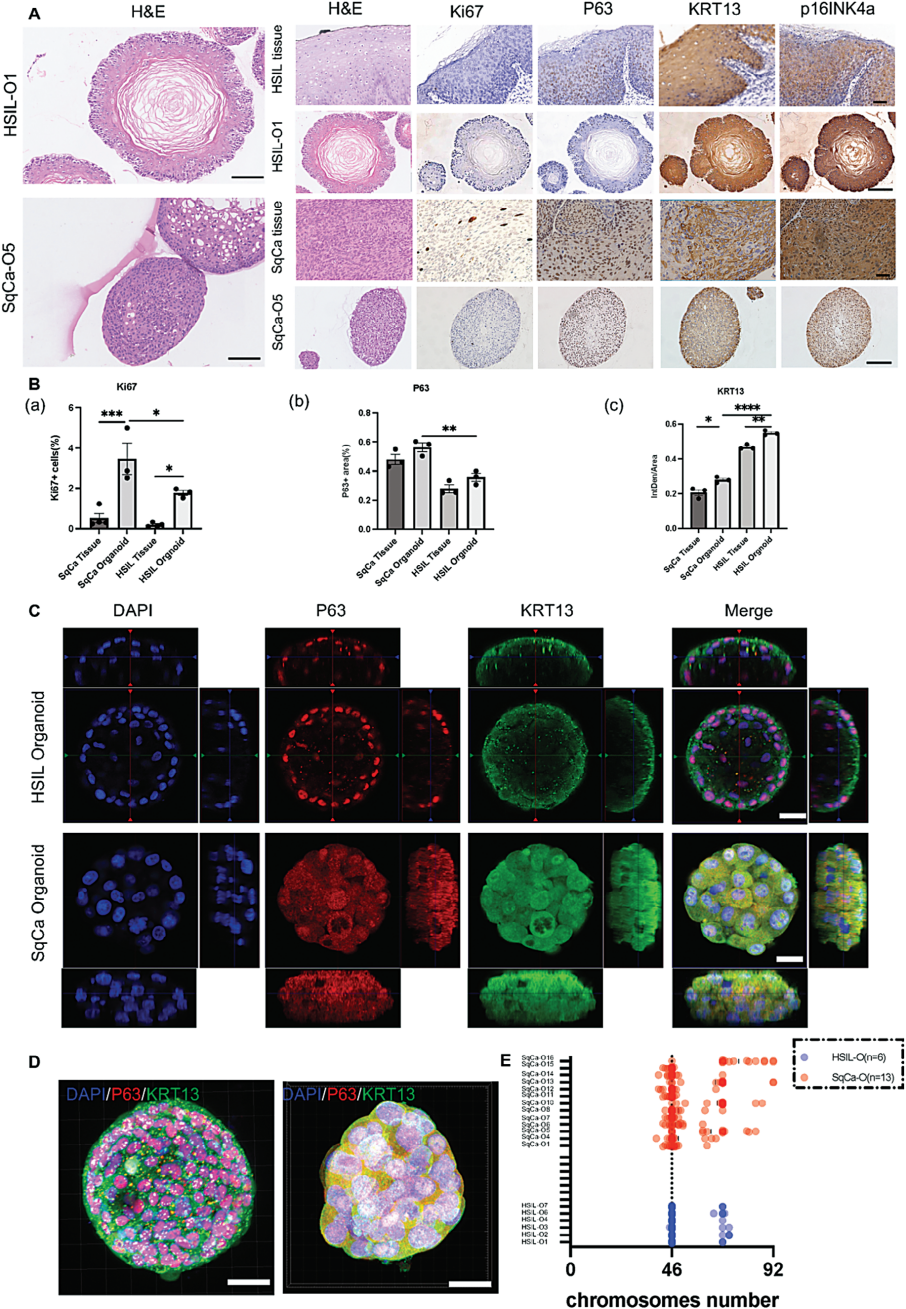
Cervical pre‐tumoroids and tumoroids reproduce the primary lesion and differ in 3D structure. A) H&E staining and positive immunostaining for Ki67, P63, KRT13, and p16INK4a of paraffin‐embedded HSIL/SqCa organoids and corresponding tissue. Scale bars, 50 µm (line 1, 3) and 100 µm (line 2, 4, and enlarged H&E diagram on the left). B) An analysis of the quantification of a) Ki67+ and b) P63+ cells in HSIL/SqCa organoids and corresponding tissue from three independent donors as indicated in (A). Average integrated density value of c) KRT13 in HSIL/SqCa organoids and corresponding tissue. Data were calculated from three independent donors, as indicated in (A). (bars show the mean of three technical replicates per donor. Data represent means ± SEM (*n* = 3), *P < 0.05, **P < 0.01, ***P < 0.001, ****P < 0.0001; one‐way ANOVA). C) Confocal images of HSIL and SqCa organoids immunolabeled for P63 (red) and KRT13 (green). The nuclei of organoids were stained with DAPI (blue) to view organoid cultures from three biological experiments. Scale bars, 20 µm. D) Whole‐mount 3D confocal image of cervical pre‐tumoroids and tumoroids immunolabeled for P63 and KRT13 created using imaris software. Scale bar, 20 µm. E) Scatter plot presenting chromosome number distribution and mean, based on analyzed organoid metaphase spreads.

To explore the mechanism underlying HSIL in SqCa development, we cultured organoids of HSIL/SqCa cells using a modified protocol (**Figure** **1**A).^[^ ^13^ ^]^ After obtaining HSIL/SqCa tissue from HSIL/SqCa HPV‐positive patients (Table S1, Supporting Information) during surgery, pap brush, or biopsy, respectively, HSIL/SqCa samples were pelleted into pieces. ≈70% of cervical cancer patients are positive for HPV16,18 + positive.^[^ ^14^ ^]^ The patients included in this study were at least those who had HSIL/SqCa confirmed by colposcopic biopsy; therefore, we had the highest number of patients who were HPV16, 18 positive, in addition to other high‐risk types of HPV:33, 39, 51, 52, 58, 59, and 66. Because of the different cell compositions of HSIL and SqCa tissues, we innovatively used an enzyme mixture named SIL Tissue dissociation solution (composed of 2 mg mL^−1^ collagenase I, 2 mg mL^−1^ Dispase II, and 0.2 mg mL^−1^ DNase I) to treat HSIL sample pieces, while 2 mg mL^−1^ collagenase I was used to treat SqCa sample pieces to obtain a sufficient number of cells to the maximum extent. Thereafter, the cells were seeded into the basement membrane extract (BME) matrix and submerged in the culture medium. We optimized the medium composition of both cultures to allow for long‐term expansion. Cervical pre‐tumoroids culture medium contained 10 components: Noggin, fibroblast growth factor 7(FGF‐7), epidermal growth factor (EGF), B27, nicotinamide, *N*‐acetyl cysteine, Rho kinase (ROCK) inhibitor (Y‐27632), transforming growth factor b inhibitor A83‐01, forskolin and p83 MARP inhibitor (SB202190), and cervical tumoroids could expand long‐term without EGF. Using our method, both fresh and frozen primitive cells grew well in the corresponding medium (Figure 1D), with success rates of 84.6 (22/26) and 83.3% (15/18) for cervical pre‐tumoroids and tumoroids, respectively. After culturing for 7–14 days (Figure 1B,E), the number of organoids initiated from the same number of primary cells per well was calculated, and HSIL organoids (HSIL‐O) were fewer than SqCa organoids (SqCa‐O) (*P* < 0.01; Figure 1F). The lower efficiency of HSIL‐O compared with SqCa‐O may be explained by the lack of genetic mutations and HPV integration into the host DNA.^[^ ^15^ ^]^ However, both organoids displayed substantial proliferation (Figure 1C) and long‐term expansion (HSIL‐O expanded for 80 days, and SqCa‐O for 8 months) (Figure 1C). The organoids exhibited high growth activity after cryopreservation (Figure 1C). Under contrast microscopy, both organoids were dense and solid spherical, and the larger HSIL‐O showed spherical internal depression (Figure 1C), which was consistent with the spherical internal differentiation observed in clinical hematoxylin and eosin (H&E) staining (**Figure** **2**A).

### 2.2 Cervical Pre‐Tumoroids and Tumoroids Reproduce the Primary Lesion

To better characterize the established culture systems, we analyzed the histological properties of both organoid lineages (Figure 2A–D) and compared them with their respective tissues of origin (Figure 2A). In the ectocervix, cuboidal basal cells are progenitor cells that are essential for constant tissue regeneration and express the proliferation marker Ki67, whereas the stratified squamous epithelium is characterized by the expression of cytokeratins, including KRT13.^[^ ^16^ ^]^ In addition, the transcription factor Tp63 is a master regulator essential for squamous epithelium development. Figure 2A presents a case of HSIL with more than two‐thirds of the disordered arrangement of squamous epithelial cells caused by a persistent infection with high‐risk HPV. When cervical SqCa develops, the basal membrane is breached and the layered structure is lost. Figure 2A shows a case of cervical SqCa with positive HPV test results. Cells in the whole layer of cervical SqCa tissue show atypia, including koilocytes, giant tumor cells, increased nuclear mitosis, and pathological mitosis. Although HSIL‐O showed a dense and solid morphology in culture (Figure 1B) resembling its SqCa counterparts, histological analysis revealed a striking difference (Figure 2A–E). Standard H&E staining and immunofluorescence revealed a polarized stratified multilayered architecture of HSIL‐O (Figure 2A,C), whereas an unstratified mass phenotype was observed in SqCa‐O (Figure 2A,C), both reminiscent of their respective native tissues. Certified by at least two pathologists, in contrast to cervical tumoroids, which showed loss of stratification and poor cellular polarity, cervical pre‐tumoroids displayed features of squamous differentiation; that is, the outer layer of the organoid contained disordered basal cells and the inner layer showed differentiated squamous epithelial characteristics (Figure 2A,C). Cervical pre‐tumoroids (Figure S1Aa, Supporting Information) and tumoroids showed koilocytes (Figure S1Ba, Supporting Information), indicating viral infection. Cervical tumoroids also had the characteristics of tumor cells, such as abundant mitotic figures (Figure S1Bb, Supporting Information), atypical, large, and hyperchromatic nuclei, tumor giant cells (Figure S1Bc, Supporting Information) and intercellular bridges (Figure S1Bb, Supporting Information). The expression of the proliferation biomarker Ki67 was significantly higher in SqCa‐O than that of HSIL‐O (Figure 2B(a); *P* < 0.05). In HSIL‐O, Ki67 was mainly distributed in the outer basal cell layer (Figure 2A), whereas it was expressed in the entire sphere of SqCa‐O (Figure 2A), which corresponded to the growth efficiency of the two types of organoids in Figure 1E. Staining for the common proliferation marker Ki67 confirmed that the longevity of our organoid cultures was driven by continuous stem cell proliferation and self‐renewal. Accompanied by the proliferation biomarker Ki67, the basal cell‐restrictive marker P63 was expressed in the outer sphere and scattered in the inner layer of HSIL‐O, whereas it was expressed abundantly throughout SqCa‐O^[^ ^16^ ^]^ (Figure 2A,C) and the positive area proportion of P63 in HSIL‐O was much lower than that in SqCa‐O (Figure 2B(b); *P* < 0.01). KRT13 is a differentiation marker expressed in the upper cervical base that decreases with an increase in the degree of cervical tumor malignancy. The expression of KRT13 in HSIL‐O and SqCa‐O corresponded to their source organization; that is, the mean gray value (integrated density/area) of KRT13 in SqCa‐O was lower than that in HSIL‐O (Figure 2B(b); *P* < 0.001), indicating a defect in normal squamous differentiation in SqCa‐O (Figure 2A,C). As for the common diagnostic index of cervical cancer, p16INK4a staining, both the nuclei and cytoplasm of HSIL‐O and SqCa‐O showed strong positivity (Figure 2A). To precisely reflect the real structures of the two organoids, Imaris software was also used for 3D reconstruction (Figure 2D) and Z‐stack presentation (Videos S1 and S2, Supporting Information) of immunofluorescence images stained with P63 and KRT13. It is worth mentioning that we have innovated a 3D structure staining method, the “microscopic 3D structure immunofluorescence staining method,” that is, single or multiple organoids were stained under a dissecting microscope without destroying their own 3D structures (see Experimental Section for details). This method is conducive to the reconstruction of their 3D structures and reflects the overall picture of organoids. Karyotype identification of all organoids showed that HSIL‐O chromosomes were concentrated in the diploid to triploid region, whereas SqCa‐O chromosomes were distributed throughout the ploidy region, even in the hypertriploid to hypotetraploid region, although both showed varying degrees of chromosomal instability (Figure 2E). Statistical analysis of the mean karyotype deviations in cervical pre‐tumoroids and tumors revealed a non‐random pattern and a significant increase in the severity of chromosome changes with the degree of malignancy (Figure 2E). Taken together, the pathological analysis proved that our model could reproduce primary lesions.

### 2.3 Cervical Pre‐Tumoroids and Tumoroids Have Their Own Characteristics in Microstructure and Cell Function

**Figure 3:**
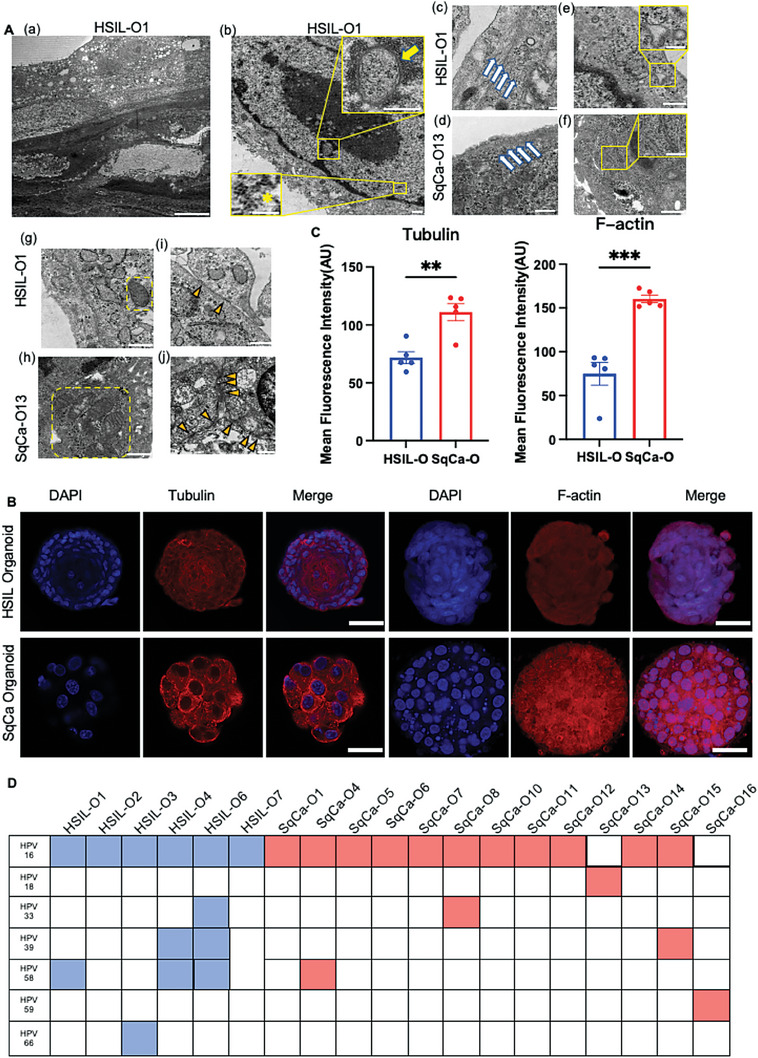
Cervical pre‐tumoroids and tumoroids have their own characteristics in microstructure and cell function. A) A TEM image revealing the microscopic morphology of HSIL‐organoids and SqCa‐organoids of HPV infection was quite different, which indicated that SqCa‐organoids were more consistent with the characteristics of cervical cancer cells. HSIL‐organoids showed a) clearly demarcated stained areas, b) intranuclear inclusion bodies (yellow arrow), naked HPV particles with diameters of 45–50 nm that differ from chromatin density (yellow asterisk), and e,f) significant endoplasmic reticulum enlargement (yellow dotted box). c,d) SqCa‐organoid showed more dense microfilaments (white arrowhead), g,h) mitochondria (yellow dotted box), and i,j) intercellular desmosome (yellow triangle). Scale bars, a) 10 µm, 500 nm (smaller insets), and b–j) 200 nm. B) Confocal images of HSIL‐organoid and SqCa‐organoid immunolabeled for microtubules (Tubulin, red) and microfilaments (F‐actin, red), respectively. Nuclei of organoids are stained with DAPI (blue). Representative organoid cultures were taken from three biological experiments. Scale bars, 20 µm. C) Mean fluorescence intensity value of invasion and migration capabilities (Tubulin and F‐actin) in HSIL‐ and SqCa‐organoids. Data were calculated from five independent donors, and representative images were indicated in (B). (Bars show the mean of three technical replicates per donor. Data represent means ± SEM (*n* = 5), **P < 0.01, ***P < 0.001; unpaired *t*‐test.) D) The detection of HPV type was performed for HSIL‐ and SqCa‐organoids. including HPV16, 18, 26, 31, 33, 35, 39, 45, 51, 52, 53, 56, 58, 59, 66, 68, 73, and 82. The table lists the subtype‐specific viral transcripts in the HSIL‐organoid (blue) and SqCa‐organoid (red) lines. The results are derived from organoids in early passages (earlier than 5 passages).

**Figure 4:**
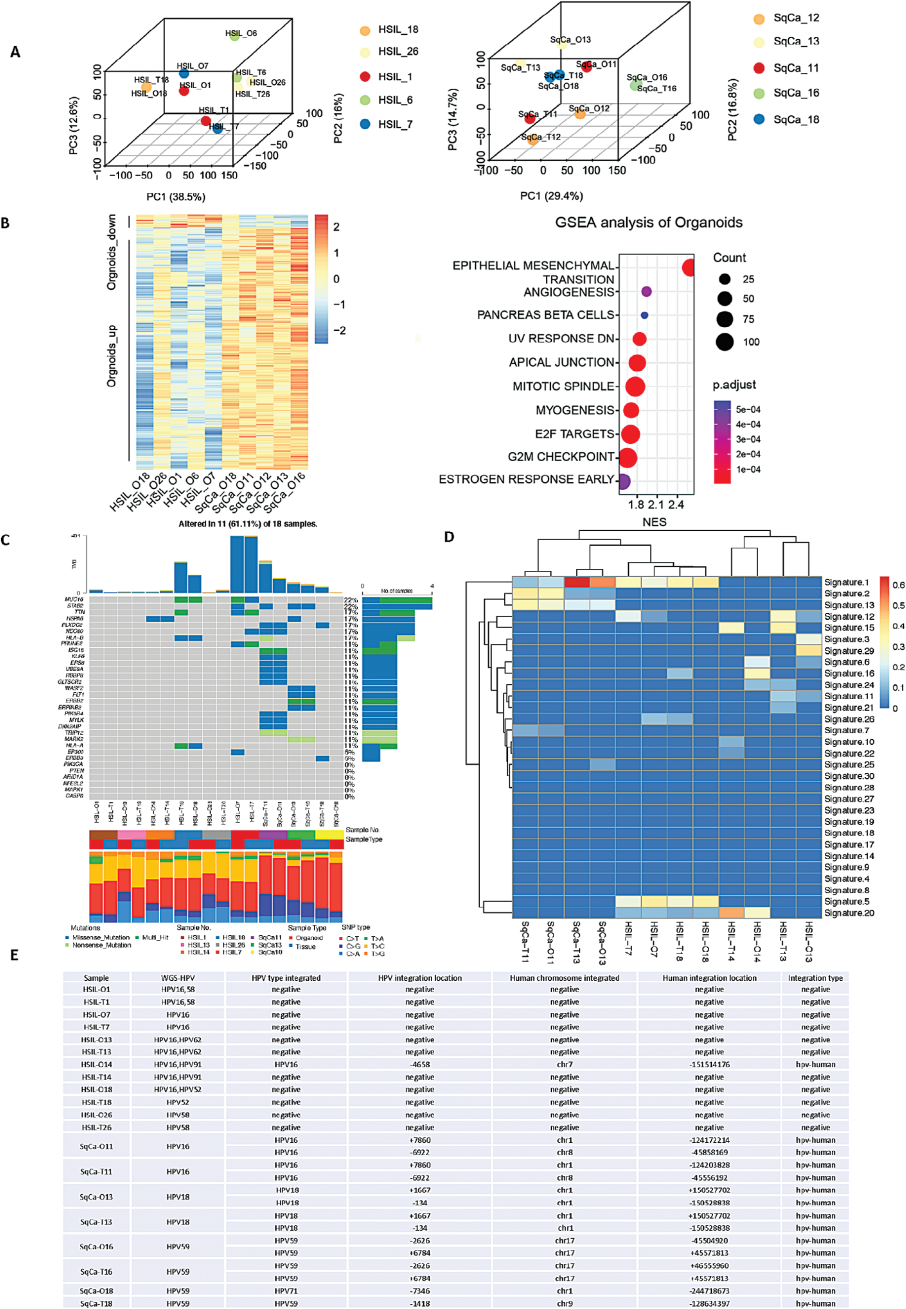
Transcriptomic and genomic analyses of cervical pre‐tumoroids and tumoroids revealed disease‐associated traits. A) PCA plot of RNA‐seq data of cervical pre‐tumoroids (*n* = 5) and tumoroids (*n* = 5) as well as their corresponding tissue. For each individual, matched tissue (T) and organoid (O) pairs are displayed. B) A heatmap and GESA cluster analysis of all differentially expressed genes as identified by RNA‐seq analysis of cervical pre‐tumoroids (*n* = 5) and tumoroids (*n* = 5). Colors range from blue (low expression) to red (high expression). C) Somatic mutations in relevant genes of cervical cancer. For each individual, matched tissue (T) and organoid (O) pairs are displayed. D) Somatic mutation signature of cervical pre‐tumoroids (*n* = 4) and tumoroids (*n* = 2) as well as their corresponding tissue. For each individual, matched tissue (T) and organoid (O) pairs are displayed. Because HSIL‐1 and HSIL‐26 mutated genes were too few, the results were not included. The official annotated database of mutational features: (https://cancer.sanger.ac.uk/signatures/sbs/) E) HPV fragment detection and HPV integration sites in cervical pre‐tumors (*n* = 6), cervical tumors (*n* = 4), and their corresponding tissues.

Given the significant difference at the histological level, we further studied the subcellular structure of cervical pre‐tumoroids and tumoroids by Transmission Electron Microscope (TEM) (**Figure** **3**A) and found that the two were distinct and the subcellular features of primary tissue were faithfully recapitulated in the two organoids. TEM revealed that the outer and inner layers of the cervical pre‐tumoroids were markedly different. The staining of the outer sphere was light, and the outer cells were arranged in cubes, whereas the staining of the inner sphere was deep, and the inner cells were flat (Figure 3A(a)). Deeper staining usually indicated increased keratin deposition (Figure 3A(a)). Cervical pre‐tumoroid HSIL‐O1 without HPV integration (**Figure** **4**E) demonstrated the presence of naked HPV particles (Figure 3A(b)), intranuclear inclusion bodies (Figure 3A(b)), and endoplasmic reticulum enlargement (Figure 3A(e)). These characteristics indicated the presence of a viral infection in the organoid. SqCa‐O showed denser microfilaments (Figure 3A(c,d)), mitochondria (Figure 3A(g,h)), and intercellular desmosomes (Figure 3A(i,j)) than those of HSIL‐O by TEM, reflecting the characteristics of SqCa‐O cancer cells at the subcellular level. To confirm the TEM results, we stained cervical pre‐tumoroids and tumoroids microtubules (Figure 3B) and microfilaments (Figure 3B). Their significant differences (Figure 3C; *P* <  0.01, *P* < 0.001) supported the TEM results. Immunofluorescence staining of the microtubules and microfilaments of cervical pre‐tumoroids was significantly weaker than those of cervical tumors, which showed that cervical tumoroids, as a cluster of tumor cells, had invasive and migratory abilities (Figure 3B,C). Human papillomavirus (HPV) infection is a leading cause of cervical cancer.^[^ ^17^ ^]^ As described in the Experimental Section, we focused on HPV typing of HSIL‐O and SqCa‐O lines using a series of probes (Figure 3D). All organoids contained viral transcripts from at least one high‐risk subtype of HPV, including HPV16, 18, 33, 39, 58, 59, and 66. HPV particles and inclusion bodies were observed in HSIL‐O1 using TEM (Figure 3A(b)), and HPV integration was not detected (Figure 4E). Thus, these results demonstrated that cervical pre‐tumoroids and tumoroids have their own characteristics in terms of microstructure and cell function and retain the same HPV type expression as the patient's original tissue.

### 2.4 Transcriptomic and Genomic Analyses of Cervical Pre‐Tumoroids and Tumoroids Revealed Disease‐Associated Traits

To assess the differences in the gene expression profiles of cervical pre‐tumoroids and tumoroids, samples were subjected to bulk RNA‐seq analysis. Principal component analysis (PCA) of cervical pre‐tumoroids, cervical tumoroids, and their originating tissues was performed to determine the extent of similarity between samples (Figure 4A). PCA showed a general clustering of cervical pre‐tumoroids or tumoroids by patient (Figure 4A) indicating the high fidelity of organoids in preserving transcriptional signatures. GSEA cluster analysis of organoid transcriptomes in the two organoid groups revealed significant differences in cervical pre‐tumoroids and tumoroids in the G2M CHECKPOINT, E2F TARGETS, MITOTIC SPINDLE, SPICAL JUNCTION, ANGIOGENESIS, MESENCHYMAL TRANSITION, and other pathways (Figure 4B). This further enhanced the pre‐cancerous features of HSIL (Figure 4B). To analyze the mutational landscape of the organoid lines, we performed whole‐genome sequencing (WGS) on lines for which we were able to collect DNA from a small amount of tissue before digestion, including six HSIL cases and four SqCa cases. We obtained matched normal tissues from individuals for sequence comparison, making it possible to differentiate somatic variants. Although there were differences between samples due to the heterogeneity of different patients, overall, organoids and their corresponding tissues were consistent in the total number of mutations and the mutation spectrum (Figure 4C). In addition, the total number of HSIL‐O mutations was lower than that of SqCa‐O mutations, which is consistent with the difference between pre‐cancerous tissues and cancer. Surprisingly, for all lines, we identified unique and individual‐specific mutational profiles that were largely conserved between tissues and respective organoids (Figure 4C). The number of mutations associated with HSIL‐O was small, consistent with the characteristics of its pre‐cancerous tissue origin. HSIL‐O7 was the only one with more mutations, but was not tumor‐related. The mutational signatures maintained a one‐to‐one correspondence between the organoids and their paired tissues (Figure 4D). For example, HSIL‐7 was significant for signatures 1, 5, 12, whereas SqCa‐11 was significant for signatures 2 and 13, of which signatures 2, 5, and 13 have been found in cancer types and signatures 2 and 13 seem to be the most common in cervical and bladder cancers (Figure 4D). Moreover, signatures 2, 13 have been attributed to the activity of *APOBEC1, APOBEC3A*, and/or *APOBEC3B* cytidine deaminases. Because the number of mutations in the pre‐cancerous lesion HSIL‐1 was too small, the calculated mutation signatures were not accurate; therefore, they were not listed. HSIL‐O7 and HSIL‐O18 derived line harbored mutations in the tumor suppressor gene *MUC16*, which has recently been reported to be mutated in cervical cancer.^[^ ^18^ ^]^ Additionally, the HSIL‐O7 cell line showed evidence of alterations in PRUNE2, which plays multiple roles in cell cycle progression, cytokinesis, and apoptosis (Figure 4C). In contrast, the mutated genes in SqCa‐derived tumors involved common targets, such as *HLA‐B*,^[^ ^19^ ^]^ *ERBB3*,^[^ ^20^ ^]^ *ERBB2*,^[^ ^21^ ^]^ *NDC80*,^[^ ^22^ ^]^ *KLF6*,^[^ ^23^ ^]^ *EPS8*,^[^ ^24^ ^]^ *WASF2*,^[^ ^25^ ^]^ *MARK2*,^[^ ^26^ ^]^ and *PIK3R4* ^[^ ^27^ ^]^ and genes in the DNA repair pathway, such as *TRIP12* ^[^ ^28^ ^]^ and *CDKN2AIP*, which showed high levels of concordance with the previously identified recurrently mutated genes (Figure 4C). HPV‐related genes, such as *ISG15*,^[^ ^29^ ^]^ *FLT1*,^[^ ^30^ ^]^ *UBE3A*,^[^ ^31^ ^]^ *SERPINB2*,^[^ ^32^ ^]^ and *RBBP8* ^[^ ^33^ ^]^ were also mutated, which is consistent with the role of HPV in cervical SqCa (Figure 4C). In the majority of cases, the organoids displayed higher enrichment in variant allele frequency (VAF) compared to the respective tissues, reflecting the enrichment of target cells in our culture system, whereas the primary tissue often contained other noncancerous cell types, such as blood cells and/or stromal components (Figure 4C). The integration of HPV DNA into the host genome is considered one of the most critical risk factors for cervical carcinoma development.^[^ ^34^ ^]^ The level of HPV integration is positively correlated with cervical intraepithelial neoplasia (CIN) grade and is recommended as a marker of disease progression.^[^ ^35^ ^]^ Because viral oncogenesis is mostly facilitated by viral gene integration, we also analyzed viral integration patterns in the sequenced organoids. Interestingly, HPV16 integration on chr7 chromosome was detected in HSIL‐O14, but no HPV integration was detected in its corresponding HSIL‐T14, which may be because HPV integration occurred in organoids during culture or HPV integration did not occur in the HSIL tissues submitted for examination (Figure 4E). The analysis revealed that the HPV phenotype and integration sites of the organoids and their source tissues were highly consistent, indicating that our model retained the characteristics of the HPV genome (Figure 4E).

### 2.5 Cervical Pre‐Tumoroids and Tumoroids Recapitulate Corresponding Disease Phenotype In Vivo

**Figure 5:**
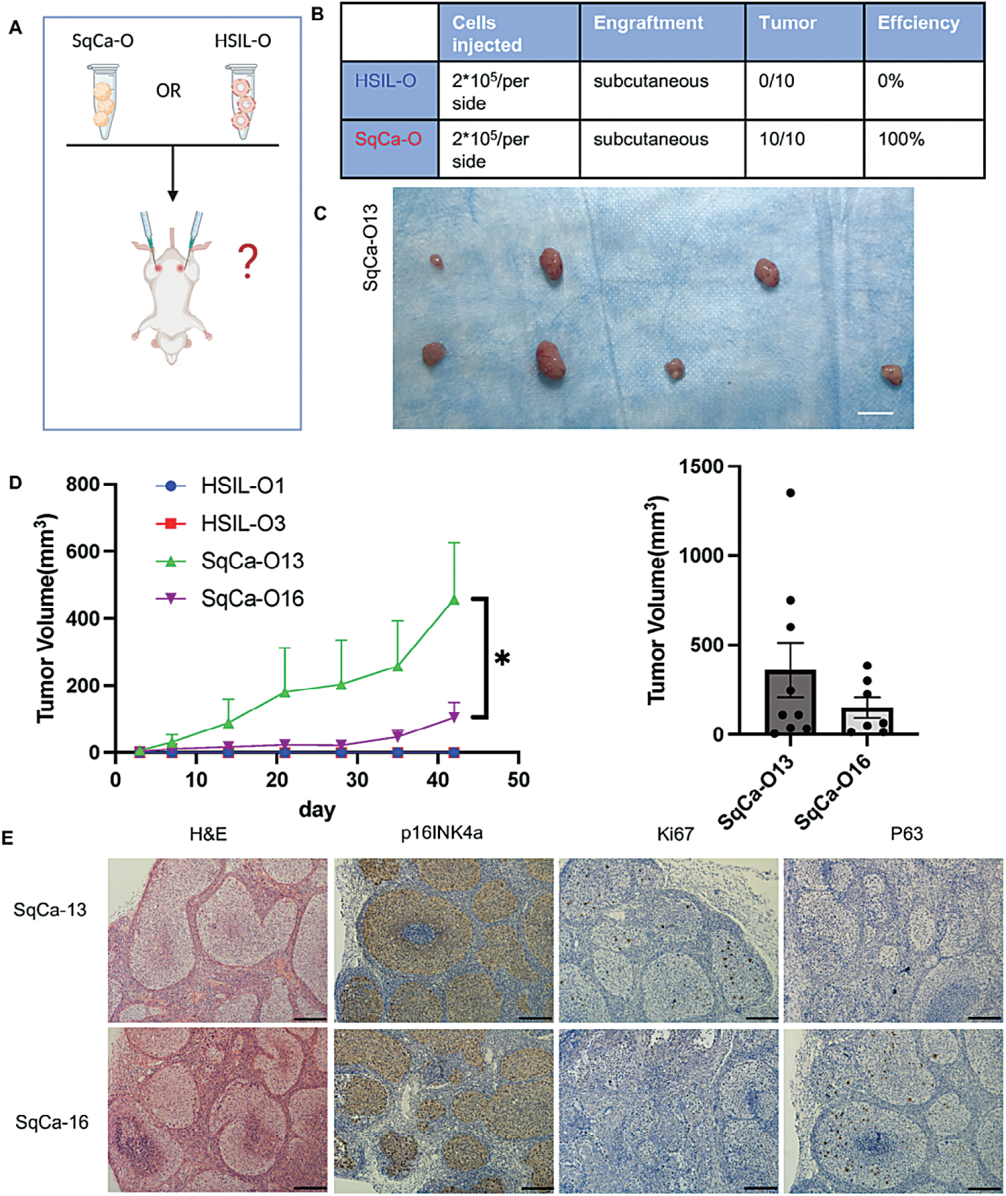
Xenografted cervical pre‐tumoroids and tumoroids recapitulate the pathohistological characteristics of cervical tumors. A) Experimental design. HSIL‐organoid or SqCa‐organoids expanded for >1 month in culture were transplanted subcutaneously (SC) of BALB/C nude mice and analyzed for tumor growth. B) Tables summarizing the number of cells and sites of engraftment. No tumor lesions were found in any of the mice injected with HSIL‐organoids. C) Significant tumor growth was observed in the mice bearing SqCa‐organoids after being transplanted subcutaneously 2–3 months, Scale bar, 1 cm. D) Tumor volume changes after organoids injection were continuously monitored. The days represent post‐implantation of organoids cells. No tumor lesions were found in mice injected with HSIL‐organoids (bars represent means ± SEM, *P < 0.05; unpaired *t*‐test). E) Representative histological overview images of tumors derived from subcutaneous injections with each organoid line. H&E, p16INK4a, Ki67, and P63 staining are shown. Scale bars, 100 mm.

To test the disease phenotype of the two organoids, we subcutaneously injected the HSIL‐O and SqCa‐O lines (**Figure** **5**A) into immunodeficient mice. Organoids from four independent lines, SqCa‐O13, SqCa‐O16, HSIL‐O1, and HSIL‐O3, were injected into the right and left flanks of five immunodeficient female mice (*n* = 5 mice/10 locations in the two groups) (Figure 5A). 3months after injection, the two SqCa‐O lines (SqCa‐O13 and SqCa‐O16) demonstrated 100% xenotransplantation success (Figure 5B). All mice injected with SqCa‐O13 developed tumors; two mice developed tumors on both flanks, and the other three developed tumors on the left or right flank (Figure 5C). Tumor formation in mice injected with SqCa‐O16 was similar to that in mice injected with SqCa‐O13 (Figure S2A,B, Supporting Information). However, the HSIL‐O lines (HSIL‐O1 and HSIL‐O3) remained localized, with lower proliferation and no obvious outgrowth (Figure 5B) after 3 months. To investigate the disintegration process of cervical pre‐tumoroids in immunodeficient mice, we re‐injected HSIL‐O3 and found that from days 2 to 15, the organoids gradually disintegrated, the BME used for localization gradually disintegrated, and the injection site was surrounded by interstitial components (Figure S2C, Supporting Information). We speculated that HSIL‐O was still in a pre‐cancerous state, which, combined with the immunohistochemical results for Ki67 (Figure 2A), could explain the low tumorigenic potential. The growth efficiencies of the different organoid lines differed, and the growth rate of SqCa‐O13 was significantly higher than that of SqCa‐O16 (Figure 5D, *P* < 0.05). Xenotransplantation of the SqCa‐O line recapitulated the histological and molecular features of the primary tumor (Figure 5E). Occasional superficial extensions into the adjacent stromal tissue were observed, suggesting an invasive nature. The distribution of carcinoma nests of different sizes was observed, with necrosis in the center and a moderate intensity of Ki67 (Figure 5E). All tumors were positive for the surrogate marker of viral infection p16INK4a and the basal cell‐restrictive marker p63 (Figure 5E).

### 2.6 Cervical Pre‐Tumoroids and Tumoroids Demonstrate Patient‐Specific Drug Responses

**Figure 6:**
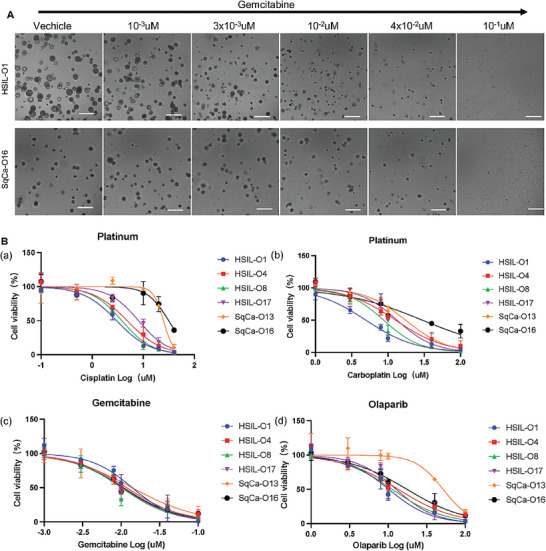
Cervical pre‐tumoroids and tumoroids show patient‐dependent drug responses and potential of mechanism research. A) Representative bright‐field images of gemcitabine‐treated HSIL‐ and SqCa‐organoids. Scale bar, 100 µm. B) Representative dose‐response curves for a) cisplatin, b) carboplatin, c) gemcitabine, and d) olaparib. Organoids showed drug sensitivity similar to that of the patients. SqCa‐organoids were more resistant to platinum than HSIL‐organoids. Dots represent the mean of technical replicates. Error bars represent the SD of technical replicates (*n* = 3).

Surgical excision using cold knife conization, laser conization, or the loop electrosurgical excision procedure (LEEP) is the gold standard treatment for HSIL.^[^ ^36^ ^]^ Positive HPV, abnormal cytology, and positive endocervical margins are high‐risk factors for HSIL recurrence within 24 months of LEEP in patients with HSIL.^[^ ^37^ ^]^ Recurrent disease has been reported following LEEP at a cumulative rate of 14% in women with CIN3 and 9% in women with CIN2^[^ ^38^ ^]^ over a 6‐year period. As the progression from HSIL to cervical cancer takes a long time, there is a good window for intervention in the progression of HSIL. Recently, multiple studies have shown that organoids have predictive value for cancer therapy.^[^ ^11^, ^13^, ^39^ ^]^ To explore whether HSIL‐O and SqCa‐O are amenable to drug screening,^[^ ^9^, ^40^ ^]^ we tested four standard chemotherapeutic compounds (cisplatin, carboplatin, gemcitabine, and olaparib) (**Figure** **6**B). Organoids were recovered from BME and dissociated. A total of 2500 cells were seeded in 96‐well plates and allowed to form organoids for 2 days. Then, the drugs were added at various concentrations, and viability was measured after 5 days. Cervical pre‐tumoroids and tumoroids showed patient‐specific drug responses. For example, SqCa‐16 showed the highest resistance to two platinum drugs (Figure 6B(a,b)), whereas it was highly sensitive to gemcitabine (Figure 6A,B(c)), which was consistent with the treatment of the corresponding patient. Notably, SqCa‐16 developed as a bilateral lower ureteral tumor invasion and metastasis after four cycles of TC therapy (Table S1, Supporting Information), which showed exceptionally low sensitivity to paclitaxel and platinum drugs. This was highly consistent with the drug sensitivity test results for the organoids, indicating that the organoids could quickly and accurately reflect the drug sensitivity of patients. Taken together, the application of organoid technology could help patients with drug resistance find more sensitive chemotherapeutic drugs and improve their treatment effects. As expected, HSIL‐O generally showed higher sensitivity to all four drugs than SqCa‐O (Figure 6B).

### 2.7 Cervical Pre‐Tumoroids and Tumoroids Well Retain the Immunogenicity of the Original Disease

**Figure 7:**
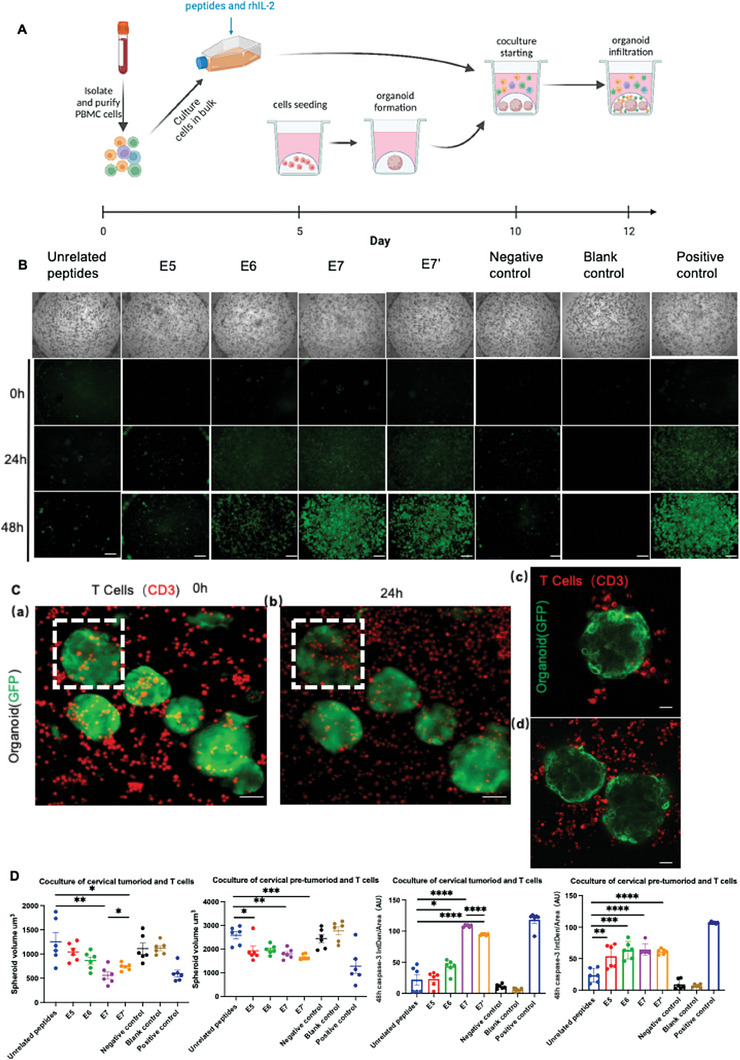
Immune infiltration and activation in organoids led to cell apoptosis and spheroid destruction. A) Scheme of the coculture protocol of organoids and PBMCs activated by peptides from healthy donors. B) Cervical tumoroids SqCa‐O11 were cocultured with or without PBMCs activated by peptides. The spheroid volume and the dynamic cleavage of caspase‐3 in the spheroids were monitored using microscopic pictures for 48 h. E5 represents the E5^15‐23^ peptide F9L (FLLCFCVLL) panel, E6 represents the E6^87‐95^ C9L (CYSLYGTTL) panel, E7 represents the E7^49‐57^ R9F (RAHYNIVTF) panel, and E7’ represents the E7^11‐20^ Y9T (YMLDLQPETT) panel. The negative control represents the coculturing organoids with PBMCs without peptides. The blank control represents the cultured organoid alone. The positive control represents the coculturing organoids with PBMCs from cervical cancer. Scale bars, 100 µm. C) Activated‐T cells targeting cervical pre‐tumoroids and tumoroids. Fluorescence microscopy analysis of luciferase/GFP expressing organoids (green) and anti‐CD3 T cells (red). (a) and (b): Exemplary data with GFP‐expressing organoids (green) and anti‐CD3‐labeled T cells (red) at 0 and 24 h of co‐culture. White boxes indicate organoids tracked in (a) and (b). (c) and (d): Exemplary data with activated T cells tageting organoids observed during live cell coculture. Scale bars, 50 µm. D) Cervical tumoroids SqCa‐O11 and cervical pre‐tumoroids HSIL‐O1 were cocultured with or without PBMCs activated by peptides. The spheroid volume and the dynamic cleavage of caspase‐3 in the spheroids were monitored using microscopic pictures at 48 h. *n* = 6 independent experiments. (* *P* < 0.05; ** *P* < 0.005, *** *P* < 0.001, **** *P* < 0.0001; one‐way ANOVA).

To expand the application of the culture platform, organoids were cocultured with peripheral blood immune cells (PBMCs) activated by HPV‐associated antigenic peptides from healthy donors, who were HLA‐A2, to screen for the antigenic peptides with the best immune effect (**Figure** **7**A). We seeded cervical pre‐tumoroids and tumoroids, and then were cocultured with PBMCs after 10 days of stimulation with HPV antigenic peptides. We explored the functional consequences of immune cell infiltration and cytotoxicity in organoids using several methods. We first examined the organoid integrity by monitoring their volume using picture‐based measurements (Figure 7B,D). Organoids cocultured with peptide‐activated PBMCs were smaller than the control spheroids at 48 h, suggesting that immune cells disrupted the organoid structure (Figure 7B,D). To examine the cause of this disruption, we assessed the global activation of caspase‐3 in the organoids using live imaging (Figure 7B,D). These complementary analyses showed that organoid destruction was correlated with active apoptosis in organoid cells involving caspase‐3 cleavage. For the quantitative monitoring of T cell‐mediated cytotoxic activity against 3D organoids, we sought to develop assays that do not require single‐cell digestion. The firefly luciferase reporter has been described as a convenient readout for viable cells following cytotoxicity in 2D cell lines.^[^ ^41^ ^]^ To adapt to this strategy, luciferase/GFP (green fluorescent protein) was introduced into the organoids using lentiviral transduction. After an initial period of 24 h, a steady increase in cytotoxicity was observed in activated T cells (Figure 7C(c,d)) and target cell lysis significantly increased in the case of activated T cells (Figure 7C(a,b)). More importantly, cervical pre‐tumoroids and tumoroids behaved distinctly when cocultured with different HPV antigenic peptides. For cervical tumoroids, E7^49‐57^ R9F (RAHYNIVTF) showed the strongest killing effect, followed by E7^11‐20^ Y9T (YMLDLQPETT). E5^15‐23^ peptide F9L (FLLCFCVLL) showed the weakest killing effect, indicating that in the late stage of cervical cancer with integrated HPV, E7^49‐57^ R9F (RAHYNIVTF) has the strongest antigenic effect, and cervical tumoroid could recognize different E7 sequences. As for cervical pre‐tumoroids, E5, E6, and E7 showed little difference in their killing effects. They all had a certain killing effect on cervical pre‐tumoroids; however, the killing effect was not as strong as that of the positive panel. This also reflected the importance of the E5 antigenic peptide for the HPV vaccine when HPV integration did not occur, which is consistent with the previous results of our research group^[^ ^42^ ^]^ (Figure S3, Supporting Information). Together, these results show that the infiltration of organoids by activated immune cells triggers cell killing and spheroid destruction, which can be enhanced by HPV antigen peptides. coculture of organoids and PBMCs can be used to screen for antigenic peptides with the most potent immune effects. More importantly, our organoid‐immune cell coculture system can recognize different antigenic peptide stimuli, demonstrating the potential of this system for immune applications.

Overall, these results indicate that by faithfully retaining the histological, transcriptomic, and genomic landscapes of their parent tissues, HSIL‐O and SqCa‐O cells can facilitate the prediction of drug sensitivity/resistance in a patient‐specific manner. Therefore, they provide an important new platform for HPV‐related cervical disease research, opening new avenues for exploring the mechanism and targeting individualized treatments for the disease.

## 3 Discussion

Recently, a large number of studies on organoids have been conducted, including bladder cancer organoids,^[^ ^43^ ^]^ breast cancer organoids,^[^ ^9^ ^]^ gastric cancer organoids,^[^ ^44^ ^]^ pre‐cancerous pathologies encompassing endometrial hyperplasia and Lynch syndrome,^[^ ^44^ ^]^ pre‐cancerous lesions of ovarian epithelial tumors,^[^ ^45^ ^]^ and gastric premalignancy.^[^ ^46^ ^]^ HPV‐related cervical diseases seriously threaten women's reproductive health and even life health. In women with molecularly detectable persistent HPV, the clinical emergence of cervical pre‐cancerous lesions is progressive. HSIL lesions typically grow slowly before invading the epithelium over several years.^[^ ^47^ ^]^ However, the determinants of invasion are still unknown. This pre‐cancerous period also provides a window of opportunity for intervention and treatment to reverse the onset and progression of cervical cancer. Human ectocervical organoids and cervical tumoroids^[^ ^13^ ^]^ have been reported; however, there are no models of cervical pre‐cancerous lesions. The lack of in vitro models that recapitulate the in vivo HSIL epithelium has hampered our understanding of HPV infection processes that occur at the cervical epithelial barrier and the key factors in the development from HSIL to invasive cervical cancer. Our study addresses this problem.

In this study, our newly established human cervical pre‐tumoroid and tumoroid models faithfully recapitulated the in vivo epithelial architecture of HSIL‐stratified squamous epithelium and the tumor cell cluster architecture of SqCa‐cancerous nests with their tissue‐specific characteristics. Cervical pre‐tumoroids and tumoroids exhibited robust expandability, captured disease heterogeneity, conserved key features of the primary tissue, including genetic background, and reproduced the lesion after in vivo transplantation.

First, we established organoids from HSIL lesions that could be expanded over the long term and showed a dense sphere profile similar to that of SqCa‐derived organoids. The most important keys to the success of organoid culture are digestion of the original tissue and composition of the culture system. During the digestion of the original tissue, it is necessary to increase the number of cells as much as possible while ensuring the viability of cells, the harsher dissociation conditions required for HSIL tissue resulting in poorer yield than cervical SqCa. Various digestion formulations were tested, including collagenase IV/I, Trypsin, Triple digestion, trypsin and collagenase, collagenase and Dispase, and collagenase, Dispase, and DNase. After selecting the combination of collagenase, Dispase, and DNase, we determined the most efficient concentration for SIL tissue digestion. And we named this combination of enzymes a “SIL Tissue dissociation solution.” For the convenience of sampling, we also tried to use pap brushes to collect HSIL cells; although the success rate was low, the corresponding organoid could be cultured. For cervical cancer tissues, we only needed to use collagenase I for mild digestion to obtain a larger number of cells because cervical cancer tissues have more tumor cell components and fewer fibrous tissue components. As for the culture system, interestingly, we did not use WNT signaling pathway potentiators such as WNT3a or RSPO1 in our cervical pre‐tumoroid and tumoroid systems, which differs from previous results,^[^ ^13^, ^16^ ^]^ perhaps indicating a weak role of the WNT signaling pathway in cervical‐derived epithelial growth. Compared with cervical tumors, there is additional EGF in the cervical pre‐tumoroid medium, which promotes the growth of epithelial cells. The cervical pre‐tumoroid structures in our study shared similarities (i.e., epithelial stratification, koilocyte appearance, nuclear atypia, and p16INK4a overexpression) with those found in patients with HSIL. These similarities indicate that cervical pre‐tumoroids structures exhibit characteristics of pre‐cancerous lesions in HSIL. Meanwhile, the differences between cervical pre‐tumoroids and tumoroids (i.e., lower expression of the proliferation marker Ki67, lower expression of the basal cell‐restrictive marker P63, and higher expression of the differentiation marker KRT13) reinforced the nature of cervical pre‐cancerous state of pre‐tumoroids. Notably, the HSIL organoids retained the capacity to self‐renew, organize, and differentiate to form mature squamous stratified organoids similar to healthy tissues^[^ ^48^ ^]^ with basal p63 + Ki67+ stem cells, p63 + Ki67‐parabasal, and p63‐ Ki67‐KRT13 + differentiated cells, whereas the SqCa organoids mainly contained P63 + Ki67 + tumor cells.

We studied and compared the subcellular structure and cell migration and invasion abilities of these two types of organoids. HPV16, 18, 33, 39, 58, 59, and 66 were readily detected in all tested lines. Our study achieved, for the first time, the establishment of HSIL organoids in a long‐term expandable natural HPV‐infected state, while strongly characterizing them by histomorphology, 3D reconstruction, and TEM. Because the special nature of HPV cannot be cultured in vitro, the pathogenesis of HPV‐related diseases and their interactions with the host are unclear. Our model can reproduce different HPV infection states, such as free and integrated HPV infections, providing a novel platform for studying the development of HPV infection and HPV‐related diseases, such as cervical cancer and head and neck tumors. Modeling the response of the epithelium to HPV infection and even carcinoma will provide information for the prevention and treatment of related diseases.

Comparative gene expression analysis of cervical pre‐tumoroids and tumoroids revealed evident changes in the transcriptomes between the two groups. Using WGS, we found that the genetic landscapes of cervical pre‐tumoroids and tumoroids were largely conserved in organoids. In the process of WGS, we also found some deviations between organoids and their source tissues, which may have been caused by the presence of other cells in primary tissues extracted from DNA or culture conditions that may favor or hinder the growth of specific mutant subclones. The number of common integration sites and the proportion of integration of cervical pre‐tumoroids and tumoroids were consistent with their corresponding source tissues, indicating that our model is in an environment of HPV infection.^[^ ^49^ ^]^

After obtaining abundant morphological and genetic evidence, we verified the function of this organoid model as a platform for functional studies. Our study showed that the resistance of SqCa‐O13 and SqCa‐O16 to cisplatin reflected the clinical response of patients to treatment. Cervical pre‐tumoroids also show patient‐specific drug responses, thereby providing conceptual evidence that organoids are amenable to (personalized) drug screening in clinical practice. Our cervical pre‐tumoroid and tumoroid models provide a tool to identify pathogenic mechanisms and target therapies for HPV‐related cervical diseases. In addition, the effective recognition of different antigenic peptides by organoids in the coculture of organoids and immune cells shows a bright future for the application of this model in immunotherapy and immunologic mechanism research. Our study represents the beginning of an extended biobank across pre‐cancerous and cancerous cervical cancers, providing promising research models and discovery tools. The organoid model overcomes the one‐size‐fits‐all treatment plan, which can predict the effectiveness and safety of different treatment methods in vitro in advance, provide pretreatment data for precision diagnosis and treatment, and effectively combine empirical and evidence‐based medicine. This may be a breakthrough in diagnosis and treatment.

There are some limitations in our study, such as the lack of organoids corresponding to CIN1 (LSIL) and the small sample size, which need to be increased for further research. We are validating this model in further research, including testing whether CRISPR‐Cas9 technology can be used in organoid applications to strengthen the study of disease mechanisms. A major advantage of the human organoid culture system is its accessibility. It facilitates the monitoring and modification of cellular activities that rely on dynamic processes in a multicellular tissue context, such as cell migration, vesicle trafficking, and cell metabolism.^[^ ^35^ ^]^ In summary, we established stably extensible cervical pre‐cancerous cells in the context of HPV and demonstrated their 3D structure, microstructure, and cellular function, which were consistent with their source tissues based on gene mapping, characteristic protein expression, microstructure, cellular function characteristics, drug sensitivity, immunogenicity, and tumorigenicity, and preserved the genetic characteristics of patients. Cervical pre‐tumoroids with pre‐cancerous properties are a promising new model to study the mechanisms and treatment of related diseases.

## 4 Experimental Section

### Human Tissue Collection for Organoid Culture

This study was performed with permission from the Ethics Committee of Tongji Hospital, Tongji Medical College, Huazhong University of Science and Technology (TJ‐IRB20230435). Patients scheduled to undergo research procedures for HSIL or cervical SqCa provided written informed consent prior to surgery and agreed to participate in the study. Additionally, HSIL and SqCa tissues were obtained from HSIL‐ and SqCa‐HPV‐positive patients, respectively, according to designated ethical protocols. HSIL tissues were obtained from surgical samples (3 × 3 mm) of HPV‐positive patients who were pathologically diagnosed with HSIL. During surgery, the surgeon uses Tischler‐Morgan forceps to collect tissues from the squamocolumnar junction adjacent to the location of the clinical biopsy. The SqCa tissues were obtained from surgical or biopsy samples (5 × 5 mm) of HPV‐positive patients pathologically diagnosed as Cervical SqCa. HPV testing The two most commonly used consensus primer sets, MY09/MY11 and GP5+/GP6+, were used to amplify a broad spectrum of HPV genotypes in a single reaction. Samples were also subjected to PCR using primers specific for HPV types 11, 16, 18, 31, 33, 35, 39, 45, 51, 52, 56, 58, 59, 66, and 68. In all cases, patients could withdraw their consent at any time, leading to the prompt disposal of their tissue and any derived material.

### Organoid Culturing from HSIL

After isolating, HSIL tissues were placed in Dulbecco's modified Eagle's medium (DMEM; Fisher Scientific) containing 10% FBS (Gibco) and 10× penicillin/streptomycin/amphotericin B, sterile solution (Solarbio, Cat#P7630) and transported to the laboratory at 4 °C. In order to reduce the occurrence of contamination, the tissues were immersed in 20 mL pre‐cooled (4 °C) phosphate‐buffered saline (PBS; homemade) containing 2× penicillin/streptomycin/amphotericin B, solution (Solarbio, Cat#P7630) under aseptic conditions for 5 min, and then washed with the same liquid three times. After each wash, the tube was placed on ice to allow natural settling, and the supernatant was removed as much as possible to remove impurities. Then the tissue was pelleted the tissue into pieces with ophthalmic scissors (cut to 0.5–1 mm^2^, if it was too broken, cell viability would be affected). After that, the proper amount of SIL Tissue dissociation solution was added (2 mg mL^−1^ of collagenase I (Solarbio, Cat#C8140), 2 mg mL^−1^ of Dispase II (Solarbio, Cat# D6430), and 0.2 mg mL^−1^ of DNase I (Solarbio, Cat#D8071)) in Dulbecco's modified Eagle medium (BOSTER, Cat#PYG0004) supplemented with 1× penicillin/streptomycin (Solarbio, Cat#P1400) and 10 µm Y‐27632 (MCE, Cat#HY‐10583) and digested the pieces in 37 °C for 40–60 min. During this process, the fragments were thoroughly mixed with collagenase I solution every 10 min, and the degree of digestion was observed. After the digestion was stopped, the cells were filtered with a 70 µm nylon cell strainer (homemade) and centrifuged for 5 min at 400 × *g*. If a visible red color was observed in the cell precipitate, 5 mL of Red Blood Cell Lysis Buffer (Standard Reagent, Cat#0210706) was added. The cells were then thoroughly mixed with BME (Cultrex Reduced Growth Factor BME, Type 2, R&D, 3533‐010‐02), and 5000–10 000 cells per 35 µL drop were plated vertically on the pre‐warmed 24‐well cell culture plates. Before adding the medium, the culture plate was solid in a 37 °C cell incubator for 30 min. The medium for cervical pre‐tumoroids consisted of advanced DMEM/f12 (Gibco, Cat#12634010) supplemented with 1× Hepes (Boster, Cat#PYG0019), 1× penicillin/streptomycin/amphotericin B, sterile solution (Solarbio, Cat#P7630), 1× Glutamax (Thermo Fisher Scientific, Cat#35050061), 1× Mycoplasma Elimination Reagent (Yeasen, Cat#40607ES03), 100 ng mL^−1^ Recombinant Human Noggin (PeproTech, Cat#120‐10C), 200 ng mL^−1^ Fibroblast growth factor 7 (PeproTech, Cat#100‐19), 50 ng mL^−1^ EGF (human) (PeproTech, Cat#AF‐100‐15), 1 µm SB202190 (MCE, Cat#HY‐10295), 2.5 mm Nicotinamide (MCE, Cat#HY‐B0150), 1.25 mm *N*‐acetylcysteine (MCE, Cat#HY‐B0215), 10 µm forskolin (MCE, Cat#HY‐15371), 1×B‐27 (Gibico, Cat#17504044), 10 µm Y‐27632 (MCE, Cat#HY‐10583), and 500 nm A83‐01 (Selleck, Cat#S7692). Following this protocol, cervical pre‐tumoroids were derived with a success rate of 84.6% (22/26).

### Organoid Culturing from Squamous Carcinoma of the Cervix

After the SqCa tissue was isolated and chopped, the proper amount of collagenase I solution was added (2 mg mL^−1^ of collagenase I, Solarbio, Cat#C8140), and the SqCa tissue pieces were digested at 37 °C for 20–40 min. During this process, the fragments were thoroughly mixed with collagenase I solution every 10 min, and the degree of digestion was observed. The digested cells were processed according to a procedure consistent with that described above, resuspended in BME, and then plated on the pre‐warmed 24‐well cell culture.

The medium for SqCa‐organoid consisted of advanced DMEM/f12 (Gibico, Cat#12634010) supplemented with 1× Hepes (Boster, Cat#PYG0019), 1× penicillin/streptomycin/amphotericin B, sterile solution (Solarbio, Cat#P7630), 1× Glutamax (Thermo Fisher Scientific, Cat#35050061), 1× Mycoplasma Elimination Reagent (Yeasen, Cat#40607ES03), 100 ng mL^−1^ Recombinant Human Noggin (PeproTech, Cat #120‐10C), 200 ng mL^−1^ Fibroblast growth factor 7 (PeproTech, Cat #100‐19), 1 µm SB202190 (MCE, Cat#HY‐10295), 2.5 mm Nicotinamide (MCE, Cat#HY‐B0150), 1.25 mm *N*‐acetylcysteine (MCE, Cat#HY‐B0215), 10 µm forskolin (MCE, Cat#HY‐15371), 1× B‐27 (Gibico, Cat#17504044), 10 µm Y‐27632 (MCE, Cat#HY‐10583), and 500 nm A83‐01 (Selleck, Cat#S7692). Following this protocol, SqCa organoids were derived with a success rate of 83.3% (of 15/18) success rate.

### Passaging and Freezing of Organoids

For passing (performed every 10–16 days), organoids were recovered by liquifying the BME incubated with pre‐cold organoid harvest solution (R&D, cat#1560173) for 30 min at 4 °C, and mechanical pipetting was used to ensure the maximum collection of organoids. Subsequently, the organoids were dissociated using TrypLE (Gibco, cat#12605028) at 37 °C; the mixture was centrifuged at 500 × *g* for 5 min. The fragments and cells obtained were resuspended in BME, and droplets were deposited in 24‐well plates. The plating density for organoid lineages should stay between 5000 and 10 000 cells per 30 µL drop for optimal outgrowth. The established organoids were amplified, cryopreserved for biobanking, and subjected to downstream analyses. The process of cryopreservation and resuscitation of the organoids was consistent with that of normal cell lines. The composition of the frozen deposit was 90% FBS, 10% DMSO, and 10 µm Y‐27632.

### Organoid Formation Efficiency Assay

For organoid diameter measure assay, fresh tissue was digested as described in the aforementioned methods. A total of 5000–10 000 cells were plated per 30 µL of BME drops into a 24‐well suspension plate overlayed with 500 µL of medium. After 14‐day culture, the diameter of each organoid was measured using a microscope (Olympus BX53). To assess the organoid formation efficiency, ≈1000 cells were plated into 5 µL of BME drops into a 96‐well cell culture plate. All full drops of BME obtained per patient were photographed using an Olympus BX53 microscope 14 days after plating, and all viable organoid structures were counted. Data analysis was performed using ImageJ software, and the experiments were performed with at least three biological replicates (three technical replicates per biological replicate).

### Immunohistochemistry

Tissues were fixed in 4% paraformaldehyde (PFA) (Servicebio, Cat# G1101) overnight, followed by dehydration and paraffin embedding. To prepare organoids for histological staining, the organoids were collected from the BME and kept as intact as possible. The organoids were removed from the culture medium and incubated in BME with pre‐cold organoid harvest solution (R&D, cat#1560173) for 45 min at 4 °C on a horizontal shaker (40 rpm) to liquify the BME and mechanical pipetting was used to ensure the maximum collection of organoids. The BME droplets dissolved completely. The 1 mL pipette tip was coated with 15 mL conical tubes and all plastic consumables with 1% bovine serum albumin (BSA) (Servicebio, Cat# G5001) until fixation, as this coating prevents organoids from sticking to the consumables’ inner sides. Using a coated 1 mL pipette tip the content of the culture well was gently resuspended 5–10 times and the organoids were transferred to coated 15 mL tubes. Organoids with identical identities from different wells were pooled in the same tubes. The organoids were centrifuged for 5 min at 500 × *g* and 4 °C to obtain a tight pellet without a visible layer of BME. The supernatant was carefully removed and the settled organoids were suspended in 4% PFA (Servicebio, G#1101) at room temperature for at least 15 min for fixation. The processed organoids were then wrapped in 3–5% agarose gel. Finally, the agarose gels containing the organoids were embedded in paraffin blocks. The sections were cut and hydrated before staining. Sections were subjected to H&E and immunohistochemical staining by using overnight incubation at 4 °C with antibodies raised against Ki67 (RRID:AB_302459, Abcam, Cat#ab16667, 1:400), p16INK4a (RRID:AB_10858268, Abcam, Cat#ab108349, 1:200), KRT13 (RRID:AB_2134681, Abcam, Cat#ab92551, 1:150), and P63 (RRID:AB_10971840, Abcam, Cat#ab124762, 1:2500). For most antibodies, antigen retrieval was performed in a citric acid solution (pH 6.0), except for the P63 antibody that required TRIS/EDTA (pH 9.0) treatment. Then, the slides were washed in TBST and incubated with a secondary antibody‐HRP (HRP Goat‐anti‐Rabbit IgG(H+L); AntGene, Cat#ANT020) conjugate for 1 h at room temperature and finally developed with 3,3′‐diaminobenzidine (DAB) for 5 min, counterstained with hematoxylin. The slides were also stained in the absence of primary antibodies to evaluate nonspecific secondary antibody reactions. Images were acquired on a Slide Scan System (Teksqray, SQS‐1000) and processed using the ImageJ software.

### Immunofluorescence

The organoids were collected as mentioned in the aforementioned method, and then suspended in 4% PFA (Servicebio, #G1101) for at least 45 min at 37  °C. The microscopic 3D structure immunofluorescence staining was used to observe single or multiple organoids and subsequent 3D reconstruction. For cervical tissue marker protein staining, the organoids were permeabilized with 0.1% Triton X‐100 (Solarbio, #T8200) for 10 min and washed three times in 1% BSA and blocked for 1 h in 1% BSA. Then, they were incubated overnight at 4  °C with primary antibodies (1:300 diluted KRT13 (Abcam, Cat#16112); 1:300 diluted P63 (Abcam, Cat#124762)), washed three times in 1% BSA, and incubated in goat anti‐mouse IgG labeled with fluorescein isothiocyanate (1:200, Proteintech, #SA00003‐1) and goat anti‐rabbit IgG labeled with cyanine 3 (1:200, Servicebio, #GB21303). Then, the organoids were incubated for 10 min with DAPI (Servicebio, #G1012). Stained organoids were mounted on a Confocal Dish (Biosharp, #BS‐20‐GJM) and examined using a Zeiss LSM980 Airyscan2 inverted confocal microscope (Zeiss).

To measurement of microfilaments and microtubules, the stock solutions of Actin‐Tracker Red‐555 (Beyotime, Cat#C2203S) and Tubulin‐Tracker Red (Beyotime, Cat#C1050) were diluted to 1:50 with 1%BSA, and organoids were stained with the resultant solutions at 37  °C for 30 min. After washing three times with PBS to remove the excessive dye, the organoids were immediately examined under a microscope with fluorescence obtained by excitation at 555 nm.

### Karyotyping

≈5–6 days after splitting of the organoids, the cultures were treated with 0.1 mg mL^−1^ colcemid (GIBCO, Cat# 15210‐040) in the culture medium for 16 h at the 37 °C in a cell incubator (with 5% CO2). Organoids were collected and dissociated into single‐cells using TrypLE. Hypotonic shock was induced by the dropwise addition of pre‐warmed 75 mm KCl and incubated at 37 °C for 10 min. The swollen cells were fixed by slow dropwise addition of ice‐cold methanol: acetic acid (3:1) while gently tapping the cell suspension. Following three rounds of fixation and washing steps, the cell suspensions were dropped on a glass slide from a height of at least 1 m, air‐dried, and mounted with DAPI‐containing Vectashield (Vector Laboratories, Cat# H 1500‐10). The slides were imaged using an Olympus BX53 microscope with a 100× objective lens and quantified by manual chromosome counting. At least 15 spreads were analyzed per organoid line.

### TEM

Briefly, organoids were removed from the Matrigel and sequentially fixed in glutaraldehyde and osmium tetroxide/potassium ferrocyanide. The organoids were dehydrated, embedded, and placed in an oven at 60 °C for polymerization for 48 h. The resin blocks were sliced using an ultrathin sectioning machine at 60–80 nm, and the slices were retrieved from a copper mesh with a 150‐mesh Fang Hua film. The copper mesh was stained in 2% uranyl acetate‐saturated alcohol solution for 8 min, washed three times with 70% alcohol, washed three times with ultrapure water washed three times, and a 2.6% lead citrate solution was used to stain for 8 min avoiding carbon dioxide, washed three times with ultrapure water, and slightly blotted dry. Copper mesh sections were placed in a copper mesh box and dried at room temperature overnight. The 70 nm sections were analyzed using a Hitachi H‐7650 transmission electron microscope.

### Bulk RNA‐seq Analysis

For RNA‐seq analysis, RNA was isolated from organoids and tissues using the RNeasy Mini Kit (QIAGEN, Cat# 74104) following the manufacturer's instructions, including DNase I treatment. Sequencing was performed on the DNBSEQ Platform (MGI Tech Co., Ltd.). Briefly, the rRNA was hybridized with the probe and digested with RNase H. cDNA synthesis, end‐repair, A‐base addition, and ligation of the index adapters were performed as follows: the first‐strand DNA was obtained through the reverse transcription of RNA and used as a template to synthesize second‐strand DNA to obtain dsDNA fragments; the dTTP‐tailed adaptor was ligated to both ends of the dsDNA fragments; the ligation products were amplified by PCR and circularized to obtain a single‐stranded circular (ssCir) library. Finally, the ssCir library was amplified using rolling circle amplification (RCA) to obtain DNA nanoballs (DNB), loaded to flow cells, and sequenced using the DNBSEQ Platform (MGI Tech Co., Ltd.) with a read length of 100 bp. Raw sequencing reads containing rRNA were filtered by mapping the sequencing reads to the rRNA database using Bowtie2 (http://bowtie‐bio.sf.net/bowtie2). The paired‐end reads were further filtered using SOAPnuke for 1) containing adaptor; 2) low‐quality bases (>50% bases with quality <5); 3) more than 10% of unknown bases; 4) trimming 15 bp of the read's head. Bowtie2 was used to map the RNA reads to the human reference genome (GCF_000001405.39_GRCh38.p13). The expression levels of all genes and isoforms were estimated using RSEM (v3.0) with the default parameters. Sequencing services were provided by BGI Genomics Co. Ltd. and Bioyi Biotechnology Co. Ltd. Wuhan, China.

### Whole‐Genome DNA Analysis

Genomic DNA from four samples was purified for ≥30×‐coverage paired‐end sequencing. The DNA was fragmented using a Covaris E‐210 ultrasonicator. By optimizing the shearing parameters, DNA fragments were concentrated at a peak of 500 bp in the relevant libraries. These fragments were purified, blunted, polyadenylated, and ligated to adaptors. After size selection by gel electrophoresis, 10–12 PCR cycles were performed. Paired‐end 90‐bp read length sequencing was performed on a HiSeq 2000 sequencer according to the manufacturer's instructions (Illumina). A bioinformatics analysis of HPV integration was conducted as described in a previous study.^[^ ^50^ ^]^

### Drug Screening

2 days prior to drug exposure, organoids were disrupted into single‐cells using TrypLE and filtered using a 70‐mm nylon cell strainer. 2000 cells were seeded in 96‐well plates and allowed to form organoids for 5 days. Various concentrations of cisplatin (MCE, Cat#HY‐17394), carboplatin (MCE, Cat#HY‐17393), gemcitabine (MCE, Cat#HY‐17026), and olaparib (MCE, Cat#HY‐10162) were added. Gemcitabine and olaparib were dissolved in DMSO. Cisplatin and carboplatin were dissolved in PBS containing 0.3% Tween‐20 (Sigma, Cat# P1379). All wells were normalized to the solvent used. The DMSO percentage never exceeded 1%, and the PBS/Tween‐20 percentage never exceeded 2%. Drug exposure was performed in triplicates for each concentration. 5 days (120 h) after drug addition, ATP levels were measured using the CellTiter‐Glo 3D Viability Assay (Promega, Cat#G9683) according to the manufacturer's instructions, and luminescence was measured using Synergy 2 (BioTek). Results were normalized to the vehicle (100%) and baseline control (staurosporine 1 mmol L^−1^ (Sigma, Cat# 62996‐74‐1); 0%).

### In Vivo Xenotransplantation Assays

Cervical pre‐tumoroid and tumoroid lines were split 3–4 days before transplantation by dissociating them into single‐cell suspensions. On the day of transplantation, small organoids were released from the BME, and a small proportion of the total sample was dissociated into single‐cells to estimate the cellular density of each sample. ≈200 000 cells were suspended in 50 µL of 50% BME/50% medium. Subcutaneous injections were performed into opposite flanks of all 5 BALB/c nude mice per line (2 flanks per mice, 200 000 cells per 50 ul per location). Mouse experiments were approved by the Ethics Committee of Tongji Hospital, Tongji Medical College of Huazhong University of Science and Technology Ethics Committee and were performed in compliance with all ethical regulations regarding animal research. Mice were sacrificed 3–4 months (90–120 days) after the injection. Tumor measurements were taken using digital calipers, and volumes were estimated using the following formula: tumor volume = (length × width^2^)/2, where length represents the largest tumor diameter and width the perpendicular tumor diameter. All tumors were subjected to immunohistochemical analysis.

### In Vitro T‐Cell Expansion

Human peripheral blood mononuclear cells (PBMC) were isolated from healthy donors and patients with cervical cancer, diluted 1:2 with PBS, and layered on a density gradient (lymphocyte separation medium, TBD science LTS10771). PBMCs were collected from the interface after centrifugation, washed with PBS, and resuspended in complete RPMI. PBMCs from healthy donors were resuspended at 1 × 10^6^ cells mL^−1^, and stimulated with 10 µg mL^−1^ peptides in ImmunoCult‐XF T Cell Expansion Media (Stemcell Technologies, Cat#10981) for 10 days at 37 °C, 5% CO_2_ with 50% media replacement every 2 to 3 days starting on day 3. The E5^15‐23^ peptide F9L (FLLCFCVLL), E6^87‐95^ C9L (CYSLYGTTL), E7^49‐57^ R9F (RAHYNIVTF), and E7^11‐20^ Y9T (YMLDLQPETT), which represent human HLA‐A*02:01‐restricted epitopes, were purchased from Sangon Biotech Co., Ltd. (Shanghai, China) and dissolved in 1× PBS at a concentration of 5 mg mL^−1^. As a control, PBMCs from cervical cancer patients were stimulated for 10 days with recombinant human IL2 (50 U mL^−1^, PeproTech Inc., Cat#200‐02), with 50% media, and recombinant human IL2 was replaced every 2–3 days starting on day 3. After 10 days, the cells were harvested.

### Coculture Protocol

Cervical pre‐tumoroids (or cervical tumoroids) spheroids were generated by seeding 1 × 10^4^ cells per well (5 µL BME) on 96‐well plates in complete respective medium. 5  days later, cocultures were started by adding 3 × 10^5^ PBMCs per well, coculture medium named complete RPMI complemented with HEPES‐containing RPMI 1640 (Thermo Fisher Scientific), 10% FBS (Gibco), 1× penicillin/streptomycin/amphotericin B, sterile solution (Solarbio, Cat#P7630), and 1× GlutaMAX (Thermo Fisher Scientific, Cat#35050061).

### Spheroid Volume Calculation

Before trypsinization, the pooled spheroids were placed in a 96‐well plate and photographed under an Olympus BX53 microscope at 4× magnification. The images were analyzed using ImageJ software by measuring the length (L) and width (W) of each spheroid. The spheroid volumes were calculated as follows: *V*  =  (*L* × *W* × *W*)/2.

### Lentiviral Transduction

To label cells with GFP and luciferase, the codon‐optimized luciferase reporter gene luc2 was amplified from the plasmid pGL4.10[luc2] (Promega, cat# E6651). Primers: F, GAACTAAACCGTCGACGCCACCATGGAAGACGCCAAAAACATAAAG; R, CCATGGTGGCGTCGACTGGTCCAGGATTCTCTTCGACATCCCCTGCTTGTTTCAACAGGGAGAAGTTAGTGGCTCCGCTTCCGGACACGGCGATCTTTCCGCCCTTC, introducing a 30 P2A sequence. The PCR fragment was inserted (as above) into the SalI site of pLV‐Pgk: GFP‐IRESPuro, 50 bp away from the GFP ORF. VSV‐G pseudotyped lentiviral particles were produced in HEK293T cells by polyethylene imine‐mediated transient transfection. 4 days after transfection, the supernatant was collected and viral particles were concentrated by ultracentrifugation (20 000 × *g*, 1 h, and 4 °C). Lentiviral transduction of organoids was performed after enzymatic dissociation into single‐cells at 37 °C for 20 min using TrypLE (Gibco, cat#12605028). Puromycin (1–2 µg mL^−1^) was added to the culture medium after 3–4 days to select for transgene‐expressing cells.

### Live‐Cell Imaging‐Based Activated‐T Cells Cytotoxicity Assay

For seeding, organoids stably expressing green (GFP) were seeded (as above) in a total volume of 5 µL per well and grown for 5 days before the addition of activated‐T cells. Activated‐T cells (prepared as above) were resuspended in a total volume of 100 µL complete RPMI per well and stained by the addition of APC‐conjugated anti‐CD3 (Biolegend, cat#553066). All live‐cell imaging experiments were performed at 37 °C, 5% CO_2_, and 20% humidity. Fluorescence images were acquired at identical positions at intervals of 6 h using Olympus BX53 and Zeiss LSM980 Airyscan2.

### Live Imaging of Spheroid Apoptosis

This was achieved by adding GreenNuc Caspase‐3 Assay Kit for Live Cells (Beyotime, Cat#C1168M) in the coculture wells and imaging green fluorescence across time using the Olympus BX53. Single pictures of each well were acquired every 6 h for 48 h and analyzed using the ImageJ software.

### Statistical Analyses and Reproducibility

The experiments described in this study were based on the analysis of at least three different organoid lines derived from three independent donors and each experiment had three technical replicates. Statistical methods were specified in the respective figure legends where applicable. Statistical analyses were performed using MS Excel and GraphPad Prism 8.0 (San Diego, CA, USA). *P*  < 0.05 was considered to be statistically significant. P values were calculated using one‐way ANOVA and unpaired Student's *t*‐test assuming a normal sample distribution, and error bars represent ± SEM. RNA‐seq and WGS data were mapped using the BWA method. The RNA‐seq data were normalized to DESeq2 and analyzed using R Studio. The WGS data‐filtering criteria are described in detail in the Experimental Section. Blinded evaluation of pre‐tumoroids, tumoroids, and their respective tissues was performed by expert pathologists.

## Conflict of Interest

The authors declare no conflict of interest.

## Author Contributions

B.H. and R.W. contributed equally to this work. B.H. was responsible for methodology, formal analysis and data interpretation, investigations, transplantations, and data curation. R.W. contributed to formal analysis and data interpretation, investigations, and transplantations. D.W. and R.L. were involved in formal analysis and data interpretation, and investigations. J.F., Z.H., and X.H. provided software and technical support. D.M. contributed to the manuscript revision. F.L. and C.S. contributed to conceptualization & resources and methodology & resources, respectively, while S.L. contributed to conceptualization, investigations, methodology, data interpretation, transplantations and resources.

## Supporting information

Supporting Information

Supplemental Video 1

Supplemental Video 2

## Data Availability

The data that support the findings of this study are available from the corresponding author upon reasonable request.
