# Supplementary material for: A Promising New Model: Establishment of Patient‐Derived Organoid Models Covering HPV‐Related Cervical Pre‐Cancerous Lesions and Their Cancers
Source: Adv Sci (Weinh). 2024 Jan 16;11(12):2302340. doi: 10.1002/advs.202302340 (PMC10966527; doi:10.1002/advs.202302340)

ADVANCED SCIENCE
Open Access

Supporting Information

for *Adv. Sci.*, DOI 10.1002/advs.202302340

A Promising New Model: Establishment of Patient-Derived Organoid Models Covering HPV-Related Cervical Pre-Cancerous Lesions and Their Cancers

Bai Hu, Renjie Wang, Di Wu, Rui Long, Junpeng Fan, Zhe Hu, Xingyuan Hu, Ding Ma, Fang Li, Chaoyang Sun* and Shujie Liao**

Supporting Information

A Promising New Model: Establishment of Patient-derived Organoid Models Covering HPV-related Cervical Pre-cancerous Lesions and their Cancers

*Bai Hu[1,2,†], Renjie Wang[1,2,†],Di Wu[1,2], Rui Long[1,2], Junpeng Fan[1,2], Zhe Hu[1,2], Xingyuan Hu[1,2], Ding Ma[1,2], Fang Li[3, *],Chaoyang Sun[1, 2,*], Shujie Liao[1, 2,*]*

Table S1 Overview of HSIL and SqCa Patients, samples information and established organoid lines

**Patient/Sample No.**	**Age**	**HPV status**	**Fresh/Cryo[a]**	**Size of sample**	**Pathological diagnosis**	**Patient Treatment**	**Organoi[illegible]**
HSIL-1	64	16,58+	Fresh	3mm*3mm	CINIII	No chemotherapy	HSIL-O1
HSIL-2	57	16+	Fresh	3mm*3mm	CINIII	No chemotherapy	HSIL-O2
HSIL-3	66	16,66+	Fresh	3mm*3mm	CINII	No chemotherapy	HSIL-O3
HSIL-4	62	16,39,58+	Fresh	3mm*3mm	CINIII	No chemotherapy	HSIL-O4
HSIL-5	48	16,31,66+	Fresh	3mm*3mm	CINII	No chemotherapy	/[c]
HSIL-6	76	16,33,39,58+	Fresh	3mm*3mm	CINII-III	No chemotherapy	HSIL-O6
HSIL-7	55	16+	Fresh	3mm*3mm	CINIII	No chemotherapy	HSIL-O7
HSIL-8	33	52+	Fresh	Pap brush	CINII	No chemotherapy	HSIL-O8
HSIL-9	30	16+	Fresh	Pap brush	CINII-III	No chemotherapy	HSIL-O9
HSIL-10	33	16+	Fresh	Pap brush	CINIII	No chemotherapy	/
HSIL-11	36	16+	Fresh	Pap brush	CINIII	No chemotherapy	/
HSIL-12	41	16,52+	Fresh	Pap brush	CINII	No chemotherapy	HSIL-O1[illegible]
HSIL-13	23	16+	Fresh	Pap brush	CINII-III	No chemotherapy	HSIL-O1[illegible]
HSIL-14	51	16+	Fresh	Pap brush	CINIII	No chemotherapy	HSIL-O1[illegible]
HSIL-15	46	16+	Fresh	Pap brush	CINII-III	No chemotherapy	/
HSIL-16	30	16+	Fresh	Pap brush	CINII	No chemotherapy	HSIL-O1[illegible]

HSIL-17	25	16,51,52+	Fresh	Pap brush	CINII	No chemotherapy	HSIL-O1[illegible]
HSIL-18	46	16,52+	Fresh	Pap brush	CINII-III	No chemotherapy	HSIL-O1[illegible]
HSIL-19	36	16+	Fresh	Pap brush	CINII	No chemotherapy	HSIL-O1[illegible]
HSIL-20	26	16+	Fresh	Pap brush	CINII	No chemotherapy	HSIL-O2[illegible]
HSIL-21	28	16,53+	Fresh	Pap brush	CINII	No chemotherapy	HSIL-O2[illegible]
HSIL-22	28	16,52+	Fresh	Pap brush	CINII	No chemotherapy	HSIL-O2[illegible]
HSIL-23	34	52,58+	Fresh	Pap brush	CINIII	No chemotherapy	HSIL-O2[illegible]
HSIL-24	28	/	Fresh	Pap brush	CINIII	No chemotherapy	HSIL-O2[illegible]
HSIL-25	44	33,58+	Fresh	Pap brush	CINII	No chemotherapy	HSIL-O2[illegible]
HSIL-26	38	58+	Fresh	Pap brush	CINII-III	No chemotherapy	HSIL-O2[illegible]
SqCa-1	33	16+	Fresh	5mm*5mm	Squamous cell carcinoma of the cervix IIIC(p）	TP(Paclitaxel+Cisplatin)	SqCa-O1
SqCa-2	60	16+	Fresh	3mm*3mm	Squamous cell carcinoma of the cervix IIa2	TP(Paclitaxel+Cisplatin)	/
SqCa-3	63	16,66+	Fresh	5mm*5mm	Squamous cell carcinoma of the cervix IIa2	TP(Paclitaxel+Nedaplatin)	/
SqCa-4	34	16,58+	Fresh	5mm*5mm	Squamous cell carcinoma of the cervix IA1	TP(Paclitaxel+Nedaplatin）	SqCa-O4
SqCa-5	56	16+	Fresh	5mm*5mm	Squamous cell carcinoma of the cervix IIB	TP(Paclitaxel+Nedaplatin)	SqCa-O5
SqCa-6	63	16+	Fresh	5mm*5mm	Squamous cell carcinoma of the cervix IIA1	TP(Paclitaxel/Docetaxel+Cisplatin)	SqCa-O6
SqCa-7	58	16+	Fresh	5mm*5mm	Squamous cell carcinoma of the cervix IIB	TP(Paclitaxel+Nedaplatin）	SqCa-O7
SqCa-8	42	16,33+	Fresh	5mm*5mm	Squamous cell carcinoma of the cervix IB2	TC（Paclitaxel+Carboplatin）	SqCa-O8

SqCa-9	56	33+	Fresh	5mm*5mm	Squamous cell carcinoma of the cervix IIIC1	TP (Paclitaxel+Nedaplatin)	/
SqCa-10	52	16+	Fresh,Cryo	5mm*5mm	Squamous cell carcinoma of the cervix IB2	TP（Docetaxel+Cisplatin）	SqCa-O1[illegible]
SqCa-11	52	16+	Fresh	5mm*5mm	Squamous cell carcinoma of the cervix IB2	TP（Paclitaxel+Nedaplatin）	SqCa-O1[illegible]
SqCa-12	73	16+	Fresh,Cryo	5mm*5mm	Squamous cell carcinoma of the cervix IIIC1	TP（Paclitaxel+Nedaplatin）	SqCa-O1[illegible]
SqCa-13	55	18+	Fresh,Cryo	5mm*5mm	Squamous cell carcinoma of the cervix IIA2	DC（Docetaxel+Carboplatin）	SqCa-O1[illegible]
SqCa-14	60	16+	Fresh	5mm*5mm	Squamous cell carcinoma of the cervix IIA1	TP（Paclitaxel+Nedaplatin）	SqCa-O1[illegible]
SqCa-15	61	16,39+	Fresh,Cryo	5mm*5mm	Squamous cell carcinoma of the cervix IB3	TP（Paclitaxel+Nedaplatin）	SqCa-O1[illegible]
SqCa-16	48	59+	Fresh,Cryo	5mm*5mm	Squamous cell carcinoma of the cervix IB3	TC（Paclitaxel+Carboplatin）, drug resistance	SqCa-O1[illegible]
SqCa-17	59	16+	Fresh,Cryo	5mm*5mm	Squamous cell carcinoma of the cervix IIICp	TP（Paclitaxel+Nedaplatin）	SqCa-O1[illegible]
SqCa-18	60	59+	Fresh, Cryo	5mm*5mm	Squamous cell carcinoma of the cervix IIIC1r	TP（Paclitaxel+Nedaplatin）	SqCa-O1[illegible]

a: Fresh: Organoids were directly established after cells were extracted from fresh original tissue.
Cryo: Organoids were established from frozen primary cells extracted from original tissue.
b: passing time: long-time (>5 passages); short-time (<5 passages).
c: Organoids were not successfully established.

Video S1: Z-stack showing the structures of different planes of HSIL-O, each containing numerous cells labeled with Krt13 (green), p63 (red), and DAPI (blue).

Video S2: Z-stack showing the structures of different planes of SqCa-O, each containing numerous cells labeled with Krt13 (green), p63 (red), and DAPI (blue).

Figure S1:

A. H&E staining of paraffin-embedded HSIL organoids. HSIL-organoids showed koilocytes (a; yellow triangles). Scale bars, 100 μm (left) and 50 μm (right).

B. H&E staining of paraffin-embedded SqCa-organoids. SqCa-organoids showed koilocytes (a; yellow triangles), increased nuclear mitosis (b; yellow asterisk), abundant intercellular bridges (b; red arrow) and tumor giant cells (c; yellow arrow). Scale bars, 100 μm (left) and 50 μm (right).

Supplement 1.

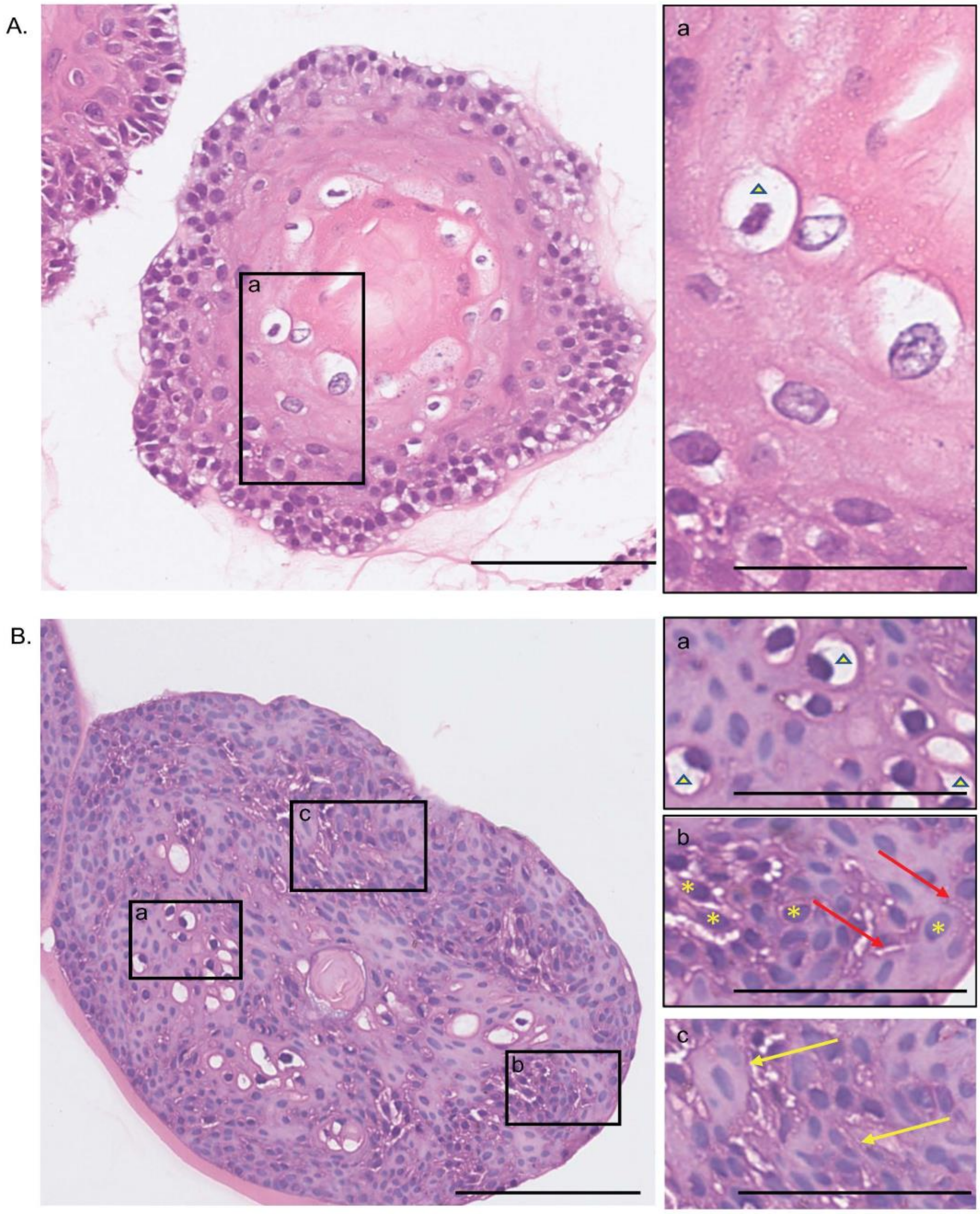

Figure S2:

A. Palpable tumor development in nude mice. Mice were killed 41 days after duplicate inoculation of tumor organoids on both left (L) and right (R) flanks. A solid tumor was evident on both side (yellow arrowheads; red dotted circle). Scale bar, 10 mm.

B. Isolated tumors. Upper panel, macroscopic views of the tumors on both sides (circle) during dissection. Lower panel, isolated tumors. Scale bar, 10 mm.

C. Microscopic changes at the injection site of HSIL-O3 in nude mice. From days 2 to 15, the organoids (yellow arrowheads) and the matrix glue used for localization gradually disintegrated, and the injection site was surrounded by nude mouse interstitial components. Scale bars, 100um.

Supplement 2.

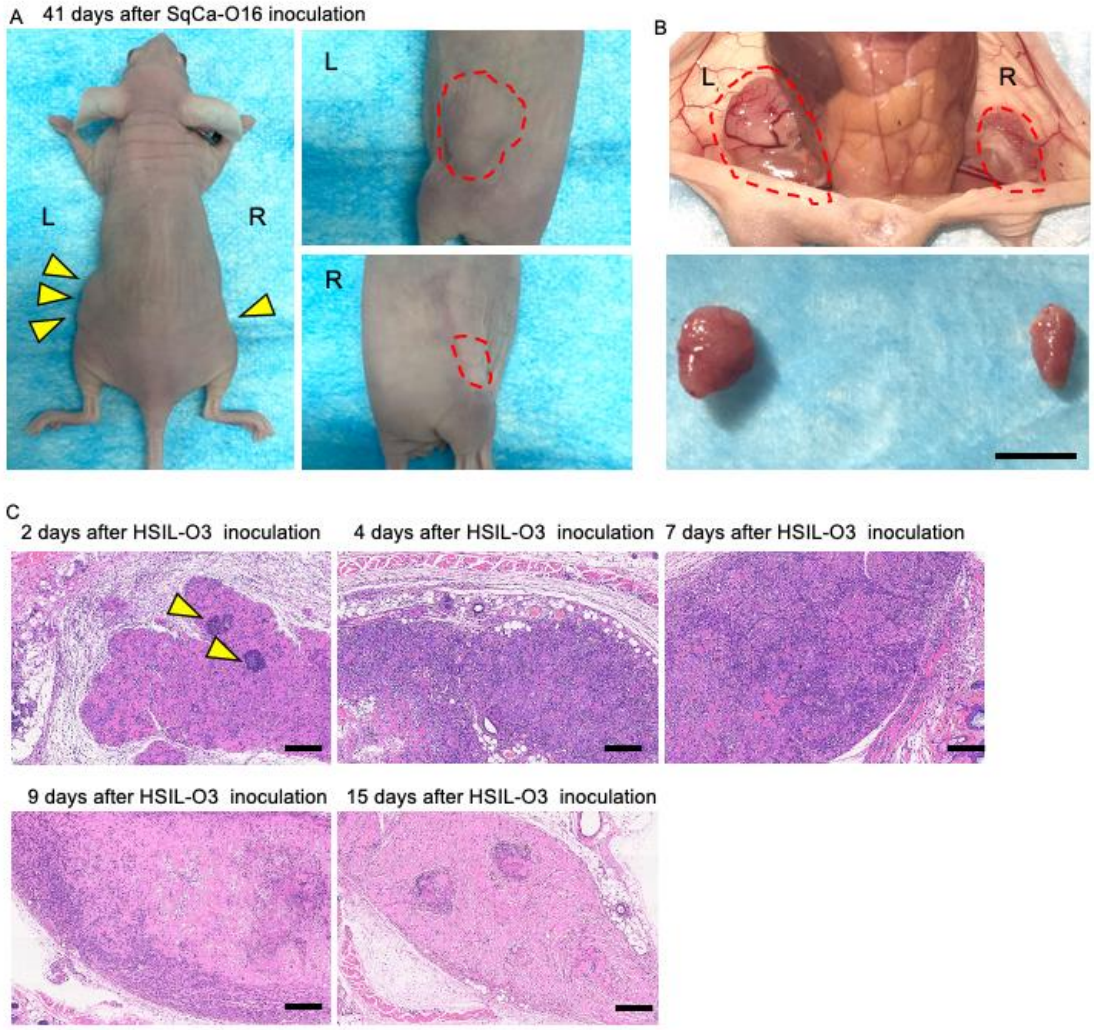

Figure S3:

Cervical pre-tumoroids HSIL-O1 were cocultured with or without PBMCs activated by peptides. We monitored the spheroid volume and dynamic cleavage of caspase-3 using microscopic pictures during 48 h. Scale bars, 100um.

Supplement 3

Cervical pre-tumoriod.

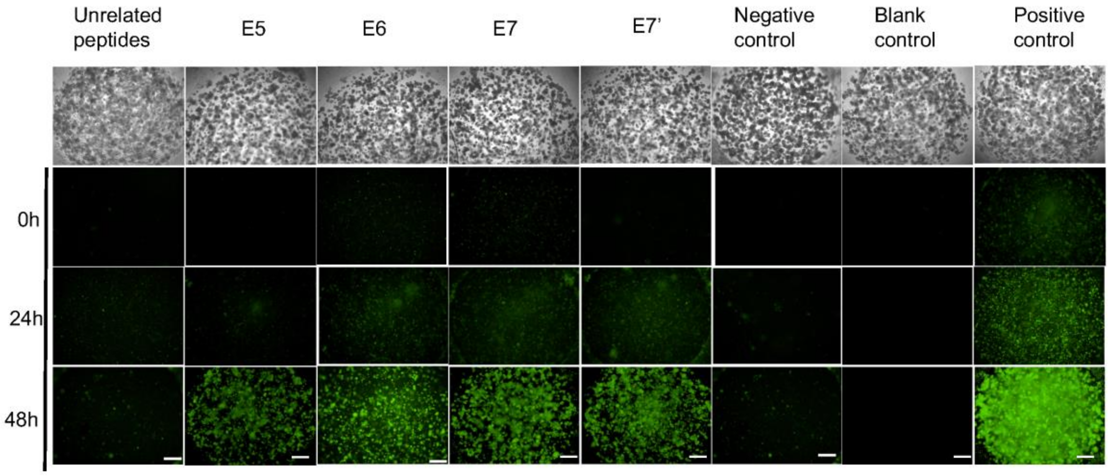

Supplement: Supplementary file 1 — Supporting Information [file ADVS-11-2302340-s003.pdf]
